# Supplementary material for: Multivariable association discovery in population-scale meta-omics studies
Source: PLoS Comput Biol. 2021 Nov 16;17(11):e1009442. doi: 10.1371/journal.pcbi.1009442 (PMC8714082; doi:10.1371/journal.pcbi.1009442)
Supplement: S1 Text — Descriptions of how the synthetic datasets are generated using SparseDOSSA for both univariate and multivariable metadata designs (with or without repeated measures) and the associated spike-in procedure to introduce feature-metadata associations. (DOCX) [file pcbi.1009442.s001.docx]

Data for differential feature model evaluations

### Synthetic null community abundances

Realistic null community data were generated using the SparseDOSSA [[1](#_ENREF_1)] (Sparse Data Observations for the Simulation of Synthetic Abundances) hierarchical model (<http://huttenhower.sph.harvard.edu/sparsedossa>). SparseDOSSA is a newly developed simulator designed to model the fundamental characteristics of real microbial communities (e.g., zero-inflation, compositionality, etc.) and to simulate new, realistic metagenomic count data with known feature-feature and feature-metadata correlations and provide a gold standard to enable benchmarking of statistical metagenomics methods, superseding previous efforts by including multiple covariates and longitudinal designs.

Briefly, SparseDOSSA’s Bayesian model captures microbial features (taxon, gene, or pathway abundances) as truncated, zero-inflated log-normal distributions, the parameters of which are hierarchically derived from a parent log-normal distribution. SparseDOSSA estimates feature-specific parameters by fitting to a real-world template dataset, and generates synthetic features from zero-inflated, truncated log-normal distributions based on both fitted and user-defined parameters on a per feature basis (**S1A Fig**). All feature-specific parameters, namely the log-mean, zero-inflation proportion, truncation point, and log-variance are empirically determined to resemble the template dataset’s properties. After sampling, the samples are rounded to the nearest integer to mimic count data. A combined dataset of the RISK [[2](#_ENREF_2)], PRISM [[3](#_ENREF_3)], pouchitis [[4](#_ENREF_4)], and NLIBD [[5](#_ENREF_5)] gut microbiomes, totaling several thousand samples, was used as empirical microbiome template data for the simulations reported in this study. To mimic realistic variation in library size, sequencing depth was generated from a lognormal distribution with average sequencing depth 50,000, resulting in approximately 30-fold to 100-fold variation in sequencing depth.

### Synthetic metadata generation

Simulated metadata matrices in simple univariate cases (UVA, UVB) were generated with continuous values from a standard normal distribution. For the univariate binary case (UVB), we additionally dichotomized the continuous variable by coding samples in the bottom and top half of the distribution as 0 and 1, respectively. For multivariate cases (MVA, MVB), we repeated the above discretization for multiple metadata by first generating from a multivariate normal distribution, and concurrently binarizing half of the metadata features at random. We considered two frequently encountered correlation structures for the multivariate cases: independent and AR (1) with coefficient 0.5, which correspond to MVA and MVB, respectively. Additionally, we considered repeated measures by incorporating random effects in these cross-sectional design matrices. To that end, we generated a simple blocking variable that is normally distributed (with mean 0 and variance 1) across subjects but invariant within subjects, representing a single random effect factor such as subject or time point (block size determined by the simulation parameters as reported in **S1A Fig**). Subsequently, we added this as an additional covariate to the fixed-effects metadata to impose correlations within the blocks, mimicking longitudinal studies. For multivariable cases (MVA, MVB), the number of covariates is fixed to 5. Similarly, for the repeated measures settings, T = 5 time points per subject is considered.

### Multivariable spike-ins of synthetic feature-metadata associations

To introduce associations between features and metadata, we used SparseDOSSA’s default additive spike-in procedure. Following Weiss et al. [[6](#_ENREF_6)], we implement the spike-ins in a balanced way across all metadata to avoid compositional bias. Briefly, SparseDOSSA standardizes both (microbial) features and metadata and randomly chooses (microbial) null features and metadata without replacement. The standardization procedure ensures that the spiked-in features are not dominated by the values of the target metadata but rather distributed similarly to the real data. Next, the standardized non-zero abundances of the selected features are modified by adding a linear combination of all spiked-in standardized metadata, in which a real-valued effect size parameter (**S1A Fig**) governs the strength of association for each associated feature-metadata pair. To create differentially abundant features, a randomly sampled fraction of 10% of the features are spiked-in to be associated with the metadata. In the multivariable case, 20% of the metadata are randomly selected to be associated with the 10% ‘differentially abundant’ features.

**References**

1. Ren BS, E; Tickle, T; Huttenhower, C sparseDOSSA: Sparse Data Observations for Simulating Synthetic Abundance. R package version 1.12.0. 2020.

2. Gevers D, Kugathasan S, Denson LA, Vázquez-Baeza Y, Van Treuren W, Ren B, et al. The treatment-naive microbiome in new-onset Crohn's disease. Cell Host Microbe. 2014;15(3):382-92. Epub 2014/03/19. doi: 10.1016/j.chom.2014.02.005. PubMed PMID: 24629344; PubMed Central PMCID: PMC4059512.

3. Morgan XC, Tickle TL, Sokol H, Gevers D, Devaney KL, Ward DV, et al. Dysfunction of the intestinal microbiome in inflammatory bowel disease and treatment. Genome Biol. 2012;13(9):R79. Epub 2012/09/28. doi: 10.1186/gb-2012-13-9-r79. PubMed PMID: 23013615; PubMed Central PMCID: PMC3506950.

4. Morgan XC, Kabakchiev B, Waldron L, Tyler AD, Tickle TL, Milgrom R, et al. Associations between host gene expression, the mucosal microbiome, and clinical outcome in the pelvic pouch of patients with inflammatory bowel disease. Genome Biol. 2015;16(1):67. Epub 2015/04/19. doi: 10.1186/s13059-015-0637-x. PubMed PMID: 25887922; PubMed Central PMCID: PMC4414286.

5. Imhann F, Vich Vila A, Bonder MJ, Fu J, Gevers D, Visschedijk MC, et al. Interplay of host genetics and gut microbiota underlying the onset and clinical presentation of inflammatory bowel disease. Gut. 2018;67(1):108-19. Epub 2016/11/02. doi: 10.1136/gutjnl-2016-312135. PubMed PMID: 27802154; PubMed Central PMCID: PMC5699972.

6. Weiss S, Xu ZZ, Peddada S, Amir A, Bittinger K, Gonzalez A, et al. Normalization and microbial differential abundance strategies depend upon data characteristics. Microbiome. 2017;5(1):27. Epub 2017/03/04. doi: 10.1186/s40168-017-0237-y. PubMed PMID: 28253908; PubMed Central PMCID: PMC5335496.
