# Supplementary material for: Multivariable association discovery in population-scale meta-omics studies
Source: PLoS Comput Biol. 2021 Nov 16;17(11):e1009442. doi: 10.1371/journal.pcbi.1009442 (PMC8714082; doi:10.1371/journal.pcbi.1009442)
Supplement: S2 Text — Details on how each of the methods compared in the Results section are implemented, run on the simulated data, and evaluated using various performance metrics. (DOCX) [file pcbi.1009442.s002.docx]

## Multivariable association test evaluation

### Preprocessing, normalizations, and transformations

We considered several commonly used normalization procedures including Total Sum Scaling (TSS), Trimmed Mean of *M*-values (TMM [[1](#_ENREF_1)]), Relative Log Expression (RLE [[2](#_ENREF_2)]), and Cumulative Sum Scaling (CSS [[3](#_ENREF_3)]) (**S1C Fig**). For TSS normalization, raw counts were converted into relative abundances by scaling each sample by the total sum (across features). For the remainder, we used the default settings of the edgeR [[4](#_ENREF_4)], DESeq2 [[5](#_ENREF_5)], and metagenomeSeq [[3](#_ENREF_3)] R packages, respectively.

In addition to the above normalization procedures, several parametric transformations were also considered. When appropriate, these variance-stabilizing transformations aim at improving parametric estimation in the presence of violated data assumptions (such as normality and homoscedasticity). These include logit and arcsine square root (AST) for TSS-normalized or proportional relative abundance data, and log transformation (**S1C Fig**). For both log and logit transformations, undefined values were replaced with zeroes (equivalent to adding a small pseudo count of 1 to the zero observations before applying the log transformation). Among other normalization/transformation methods, a ‘Default/None’ category was also considered which represents either (i) default normalization/transformation employed by the associated software or (ii) no normalization/transformation, or (iii) library size normalization in the form of a GLM offset modeling. Prior to applying any normalization and transformation, a basic filtering was performed to prune features absent in >90% of samples. As in previous benchmarking [[6-8](#_ENREF_6)], correction for multiple testing was performed using the Benjamini-Hochberg [[9](#_ENREF_9)] FDR threshold of 0.05.

Statistical methods

We selected several commonly used methods for differential abundance and multivariable association testing along with a set of experimental methods to apply on the synthetic datasets, using a combination of statistical model and normalization/transformation schemes for each method as appropriate (**S1C Fig**). All tests were conducted using the statistical software R and parallelized using custom bash scripts in a high-performance computing environment (full source code available at: <https://github.com/biobakery/maaslin2_benchmark>). The selected statistical models (abbreviations in parentheses) are as follows:

- ANCOM: following Weiss et al. [[8](#_ENREF_8)], we used the default implementation of ANCOM [[10](#_ENREF_10)] using the *ANCOM()* function call with default settings. Unlike other methods, ANCOM does not report p-values but instead returns logical indicators of whether a feature is differentially abundant based on a test statistic W. It is to be noted that in the presence of multiple covariates, ANCOM does not return statistically significant feature-metadata pairs with respect to every covariate in the model, making it infeasible for our multivariable setting. Also, we did not test the ANCOM method in repeated measures settings as it was too slow and unstable for assessment, as noted elsewhere [[11](#_ENREF_11)].
- metagenomeSeq: for the fixed effects, counts were first CSS-normalized with the default quantile supplied by the *cumNormStat()* function and the (log-transformed) CSS-normalized counts were subjected to final testing using *fitZig*() [[3](#_ENREF_3)]. For random effects, *useMixedModel was* set to TRUE in the *fitZig*() function call.
- metagenomeSeq2: same as metagenomeSeq [[3](#_ENREF_3)], except that the final testing was done using the *fitFeatureModel*() function.
- DESeq2: for fixed effects, following Thorsen et al. [[12](#_ENREF_12)], geometric means were first calculated manually from the raw counts and supplied to the *estimateSizeFactors()* function before calling the *DESeq()* function for final testing [[5](#_ENREF_5)]. Random effects modeling compatible with our setting is currently not supported by the DESeq2 software [[13](#_ENREF_13)].
- edgeR: for fixed effects, following Thorsen et al. [[12](#_ENREF_12)], normalization factors were calculated with TMM, which was followed by common and tagwise dispersion estimation steps, before invoking the standard test with the *exactTest()* function [[4](#_ENREF_4)]*.* Random effects modeling compatible with our setting is currently not supported by the edgeR software [[13](#_ENREF_13)].
- limma: the default functionality of *lmFit()* was applied to the feature counts [[14](#_ENREF_14)]. Repeated measures were handled using the *duplicateCorrelation()* function before calling *lmFit(),* in combination with appropriate normalization/transformation (**S1C Fig**).
- limma VOOM: same as limma, except that features were subjected to a voom transformation before applying limma [[15](#_ENREF_15), [16](#_ENREF_16)].
- limma2: same as limma, except that library size or scale factor is included as an additional covariate, in combination with appropriate normalization/transformation (**S1C Fig**).
- Wilcoxon: the built-in R function *wilcox.test()* using default parameters was applied to the features in combination with appropriate normalization/transformation (**S1C Fig**).
- Spearman: the built-in R function cor*.test()* was applied to the features in combination with appropriate normalization/transformation (**S1C Fig**).
- Linear model (LM): the built-in R function *lm()* with default settings was used in combination with appropriate normalization/transformation (**S1C Fig**). For random effects, we used the *lme()* function from the *nlme* R package [[17](#_ENREF_17)].
- Linear model (LM2): same as LM, except that library size or scale factor is included as an additional covariate in the model, in combination with appropriate normalization/transformation (**S1C Fig**).
- Negative binomial (negbin): we used the *glm.nb()* function from the *MASS* package [[18](#_ENREF_18)] and the *glmer.nb()* function from the *lme4* package [[19](#_ENREF_19)] for fixed and random effects respectively. In both cases, we used the logarithm of library size (for no normalization) or scaling factor (for other normalization schemes such as CSS, RLE, and TMM) as offset.
- Zero-inflated Negative Binomial (ZINB): for fixed effects, we used the *zeroinfl()* function from the *pscl* package [[20](#_ENREF_20)] with the logarithm of library size (for no normalization) or scaling factor (for other normalization schemes such as CSS, RLE, and TMM) as offset. In the absence of a robust random effect implementation of the same, the ZINB method was not considered in the repeated measures settings.
- Zero-inflated Beta (ZIB): following Peng et al. [[21](#_ENREF_21)], we used the *gamlss()* function from the R package *gamlss* [[22](#_ENREF_22)] for fixed effects and the *ZIBR()* function from the *ZIBR* R package for random effects [[23](#_ENREF_23)]. In both cases, the features are TSS-normalized before statistical testing.
- Compound Poisson (CPLM): we used the *cpglm()* function from the *cplm* package [[24](#_ENREF_24)] and the *glmmPQL()* function from the *MASS* package [[24](#_ENREF_24)] for fixed and random effects respectively. In both cases, we used the logarithm of library size (for no normalization) or scaling factor (for other normalization schemes such as CSS, RLE, and TMM) as offset. No offset was used when combined with the TSS-normalized relative counts.
- MaAsLin 1: we used the default TSS-normalized, arcsine square root-transformed linear model without gradient boosting [[25](#_ENREF_25), [26](#_ENREF_26)].
- MaAsLin 2: we used the default TSS-normalized, log-transformed linear model with half the minimum relative abundance as pseudo count.

Naming convention

The nomenclature for the model/normalization/transformation combinations for each method is described in the following set of rules:

1. For published methods with default parameters, there is no additional identifier following the name of the method, indicating default or no normalization/transformation. These include ANCOM, metagenomeSeq, metagenomeSeq2, limma, limma2, limma VOOM, DESeq2, edgeR, and ZIB.
2. Similarly, for experimental methods with custom normalization/transformation schemes, no additional identifier simply indicates either no normalization (for non-GLM methods such as LM) or library size normalization (for specific GLMs such as Negative Binomial, Compound Poisson, and ZINB).
3. Finally, for methods with additional identifiers, method name is always accompanied by a normalization scheme (after the first dot) which is followed by a transformation (after the second dot) except in cases where either no normalization or no transformation is applied. As an example, limma.CSS.LOG denotes a default limma model followed by CSS normalization and log transformation. Similarly, LM.CLR denotes a vanilla linear model followed by a CLR transformation and no normalization, whereas ZINB.TMM denotes a zero-inflated negative binomial model with TMM normalization and no transformation, and so on and so forth.

### Performance evaluation

Several performance metrics were considered for evaluation, all derived from some combination of the elements from the confusion matrix: false positives (FPs), true positives (TPs), true negatives (TNs), and false negatives (FNs). These include measures related to (i) statistical power, (ii) false discovery, and (iii) software implementation and scope, all as averages over 100 simulation runs (**S1B Fig**). Several measures were considered for statistical power - Sensitivity, Area Under the Curve (AUC), and scaled partial AUC (spAUC). The AUC was calculated as the area under the ROC curve, obtained by plotting the sensitivity versus 1-specificity for the varying p-value threshold. spAUC was calculated as the partial area over the high specificity range (0, 0.20), rescaled to mimic the interpretation of AUC (i.e., 0.5 for a random guess and 1 for a perfect classifier using p-values to discriminate between spiked and non-spiked features). The R package *ROCR* [[27](#_ENREF_27)] was used to calculate both these AUC measures. We also considered Matthew’s correlation coefficient as well as F1 scores as alternate accuracy measures of performance.

Among false discovery metrics, maximum and average of several commonly used metrics including False Discovery Rate (FDR) and False Positive Rate (FPR) were considered. When no features were declared significant (i.e., TP = FP = 0), the false discovery rate (FDR) was set to 0. Notably, Weiss et al. [[8](#_ENREF_8)] misreported false positive rate as FDR, as evident from the supplemental R code of that paper (Additional files 9 and 10 of Weiss et al [[8](#_ENREF_8)]). In order to avoid any ambiguity, we provide the analytical expressions of the above-mentioned measures (except AUC and spAUC) as follows:

$$FDR (1- Precision) =\frac{\mathrm{FP}}{FP+TP}$$

$$\mathrm{FPR}\left( 1- Specificity \right)=\frac{\mathrm{FP}}{FP+TN}$$

$$Sensitivity (Power or Recall) =\frac{\mathrm{TP}}{TP+FN}$$

$$F1 score =\frac{2TP}{2TP+FP+FN}$$

$$\mathrm{Matthew}^{'}s correlation coefficient \left( \mathrm{MCC} \right) =\frac{TP*TN-FP*FN}{\sqrt{\left( TP+FP \right)*\left( TP+FN \right)*\left( TN+FP \right)*\left( TN+FN \right)}}$$

Following Hawinkel et al. [[6](#_ENREF_6)], an alternative measure based on the p-value distribution under the null, ‘Departure from Uniformity’, was also considered. Briefly, to quantify the departures from uniformity into liberal (or conservative) direction, twice the mean distance between the diagonal line and the points in the QQ plot below (or above) the diagonal was computed. We called these measures ‘Liberal Area’ and ‘Conservative Area’, respectively. Both calculated areas are averages over all features, and they both range from 0 to 1. A combined metric called ‘Total Area’ that defines departure in either direction (defined as Total Area = Liberal Area + Conservative Area) was also computed.

Finally, we calculated computational time and convergence aspects of different methods based on their available implementation. Following Soneson and Robinson [[28](#_ENREF_28)], we record the actual time required to run each method using a single core and normalize all times for a given data set instance so that the maximal value across all methods is 1 (as reported in **Fig 1C**). Thus, a 'relative' computational time of 1 for a given method and a given data set instance means that this method was the slowest one for that particular instance, and a value of, for example, 0.1 means that the time requirement was 10% of that for the slowest method. Similarly, we estimated the ‘relative’ convergence failure rates for each method, as before, with the worst method as a reference.

**References**

1. Robinson MD, Oshlack A. A scaling normalization method for differential expression analysis of RNA-seq data. Genome Biol. 2010;11(3):R25. Epub 2010/03/04. doi: 10.1186/gb-2010-11-3-r25. PubMed PMID: 20196867; PubMed Central PMCID: PMCPMC2864565.

2. Anders S, Huber W. Differential expression analysis for sequence count data. Genome Biol. 2010;11(10):R106. Epub 2010/10/29. doi: 10.1186/gb-2010-11-10-r106. PubMed PMID: 20979621; PubMed Central PMCID: PMCPMC3218662.

3. Paulson JN, Stine OC, Bravo HC, Pop M. Differential abundance analysis for microbial marker-gene surveys. Nat Methods. 2013;10(12):1200-2. Epub 2013/10/01. doi: 10.1038/nmeth.2658. PubMed PMID: 24076764; PubMed Central PMCID: PMCPMC4010126.

4. Robinson MD, McCarthy DJ, Smyth GK. edgeR: a Bioconductor package for differential expression analysis of digital gene expression data. Bioinformatics. 2010;26(1):139-40. Epub 2009/11/17. doi: 10.1093/bioinformatics/btp616. PubMed PMID: 19910308; PubMed Central PMCID: PMCPMC2796818.

5. Love MI, Huber W, Anders S. Moderated estimation of fold change and dispersion for RNA-seq data with DESeq2. Genome Biol. 2014;15(12):550. Epub 2014/12/18. doi: 10.1186/s13059-014-0550-8. PubMed PMID: 25516281; PubMed Central PMCID: PMCPMC4302049.

6. Hawinkel S, Mattiello F, Bijnens L, Thas O. A broken promise: microbiome differential abundance methods do not control the false discovery rate. Brief Bioinform. 2019;20(1):210-21. Epub 2017/10/03. doi: 10.1093/bib/bbx104. PubMed PMID: 28968702.

7. McMurdie PJ, Holmes S. Waste not, want not: why rarefying microbiome data is inadmissible. PLoS Comput Biol. 2014;10(4):e1003531. Epub 2014/04/05. doi: 10.1371/journal.pcbi.1003531. PubMed PMID: 24699258; PubMed Central PMCID: PMCPMC3974642.

8. Weiss S, Xu ZZ, Peddada S, Amir A, Bittinger K, Gonzalez A, et al. Normalization and microbial differential abundance strategies depend upon data characteristics. Microbiome. 2017;5(1):27. Epub 2017/03/04. doi: 10.1186/s40168-017-0237-y. PubMed PMID: 28253908; PubMed Central PMCID: PMCPMC5335496.

9. Benjamini Y, Hochberg Y. Controlling the false discovery rate: a practical and powerful approach to multiple testing. Journal of the Royal statistical society: series B (Methodological). 1995;57(1):289-300. PubMed PMID: Benjamini1995.

10. Mandal S, Van Treuren W, White RA, Eggesbø M, Knight R, Peddada SD. Analysis of composition of microbiomes: a novel method for studying microbial composition. Microb Ecol Health Dis. 2015;26:27663. Epub 2015/06/02. doi: 10.3402/mehd.v26.27663. PubMed PMID: 26028277; PubMed Central PMCID: PMCPMC4450248.

11. Calgaro M, Romualdi C, Waldron L, Risso D, Vitulo N. Assessment of statistical methods from single cell, bulk RNA-seq, and metagenomics applied to microbiome data. Genome Biol. 2020;21(1):191. Epub 2020/08/05. doi: 10.1186/s13059-020-02104-1. PubMed PMID: 32746888; PubMed Central PMCID: PMCPMC7398076.

12. Thorsen J, Brejnrod A, Mortensen M, Rasmussen MA, Stokholm J, Al-Soud WA, et al. Large-scale benchmarking reveals false discoveries and count transformation sensitivity in 16S rRNA gene amplicon data analysis methods used in microbiome studies. Microbiome. 2016;4(1):62. Epub 2016/11/26. doi: 10.1186/s40168-016-0208-8. PubMed PMID: 27884206; PubMed Central PMCID: PMC29731741.

13. Cui S, Ji T, Li J, Cheng J, Qiu J. What if we ignore the random effects when analyzing RNA-seq data in a multifactor experiment. Stat Appl Genet Mol Biol. 2016;15(2):87-105. Epub 2016/03/02. doi: 10.1515/sagmb-2015-0011. PubMed PMID: 26926865.

14. Smyth GK. Linear models and empirical bayes methods for assessing differential expression in microarray experiments. Stat Appl Genet Mol Biol. 2004;3:Article3. Epub 2006/05/02. doi: 10.2202/1544-6115.1027. PubMed PMID: 16646809.

15. Law CW, Chen Y, Shi W, Smyth GK. voom: Precision weights unlock linear model analysis tools for RNA-seq read counts. Genome Biol. 2014;15(2):R29. Epub 2014/02/04. doi: 10.1186/gb-2014-15-2-r29. PubMed PMID: 24485249; PubMed Central PMCID: PMCPMC4053721.

16. Ritchie ME, Phipson B, Wu D, Hu Y, Law CW, Shi W, et al. limma powers differential expression analyses for RNA-sequencing and microarray studies. Nucleic Acids Res. 2015;43(7):e47. Epub 2015/01/22. doi: 10.1093/nar/gkv007. PubMed PMID: 25605792; PubMed Central PMCID: PMC25605792.

17. Pinheiro J. nlme: linear and nonlinear mixed-effects models. R package version 3.1-103. <http://cran> r-project org/web/packages/nlme/index html. 2012..

18. Venables WN, Ripley BD. Modern applied statistics with S-PLUS: Springer Science & Business Media; 2013.

19. Bates D, Mächler M, Bolker BM, Walker SC. Fitting linear mixed-effects models using lme4. Journal of Statistical Software. 2015;67(1). PubMed PMID: Bates2020.

20. Zeileis A, Kleiber C, Jackman S. Regression models for count data in R. Journal of statistical software. 2008;27(8):1-25. PubMed PMID: Jackman2020.

21. Peng X, Li G, Liu Z. Zero-Inflated Beta Regression for Differential Abundance Analysis with Metagenomics Data. J Comput Biol. 2016;23(2):102-10. Epub 2015/12/18. doi: 10.1089/cmb.2015.0157. PubMed PMID: 26675626; PubMed Central PMCID: PMCPMC6109378.

22. Stasinopoulos DM, Rigby RA. Generalized additive models for location scale and shape (GAMLSS) in R. 2007. PubMed PMID: Stasinopoulos2007.

23. Chen EZ, Li H. A two-part mixed-effects model for analyzing longitudinal microbiome compositional data. Bioinformatics. 2016;32(17):2611-7. Epub 2016/05/18. doi: 10.1093/bioinformatics/btw308. PubMed PMID: 27187200; PubMed Central PMCID: PMCPMC5860434.

24. Zhang Y. Likelihood-based and bayesian methods for tweedie compound poisson linear mixed models. Statistics and Computing. 2013;23(6):743-57. PubMed PMID: Zhang2013.

25. Morgan XC, Tickle TL, Sokol H, Gevers D, Devaney KL, Ward DV, et al. Dysfunction of the intestinal microbiome in inflammatory bowel disease and treatment. Genome Biol. 2012;13(9):R79. Epub 2012/09/28. doi: 10.1186/gb-2012-13-9-r79. PubMed PMID: 23013615; PubMed Central PMCID: PMCPMC3506950.

26. Morgan XC, Kabakchiev B, Waldron L, Tyler AD, Tickle TL, Milgrom R, et al. Associations between host gene expression, the mucosal microbiome, and clinical outcome in the pelvic pouch of patients with inflammatory bowel disease. Genome Biol. 2015;16(1):67. Epub 2015/04/19. doi: 10.1186/s13059-015-0637-x. PubMed PMID: 25887922; PubMed Central PMCID: PMCPMC4414286.

27. Sing T, Sander O, Beerenwinkel N, Lengauer T. ROCR: visualizing classifier performance in R. Bioinformatics. 2005;21(20):3940-1. Epub 2005/08/13. doi: 10.1093/bioinformatics/bti623. PubMed PMID: 16096348.

28. Soneson C, Robinson MD. Bias, robustness and scalability in single-cell differential expression analysis. Nat Methods. 2018;15(4):255-61. Epub 2018/02/27. doi: 10.1038/nmeth.4612. PubMed PMID: 29481549.
