# Supplementary material for: Multivariable association discovery in population-scale meta-omics studies
Source: PLoS Comput Biol. 2021 Nov 16;17(11):e1009442. doi: 10.1371/journal.pcbi.1009442 (PMC8714082; doi:10.1371/journal.pcbi.1009442)
Supplement: S3 Text — Details on differential abundance analysis of iHMP (HMP2) IBDMDB multi-omics dataset using MaAsLin 2, along with a description of the associated study design, quality control procedures, shuffle data experiments, and per-feature multivariable models for various microbial measurement types. (DOCX) [file pcbi.1009442.s003.docx]

Analysis of the iHMP (HMP2) IBDMDB multi-omics dataset

### Study design, data, and quality control

Data were obtained from the Integrative Human Microbiome Project (HMP2 or iHMP), which is described in detail in Lloyd-Price et al. [[1](#_ENREF_1)] and available through the Inflammatory Bowel Disease Multi-omics Database (IBDMDB, <http://ibdmdb.org>). Briefly, subjects included in this cohort were recruited from five academic medical centers across the US: three pediatric sub-cohorts including Cincinnati Children’s Hospital, Massachusetts General Hospital (MGH) Pediatrics, and Emory University Hospital, and two adult sub-cohorts including MGH and Cedars-Sinai Medical Center. 132 subjects were followed for one year each to generate integrated longitudinal molecular profiles of host and microbial activity during disease (up to 24 time points each; in total 2,965 stool, biopsy, and blood specimens). Self-collected stool samples were transported in ethanol fixative before storage at -80 C until DNA extraction.

Multiple measurement types were generated from many individual stool specimens, including 305 samples that contain all stool-derived measurements and 791 metagenome-metatranscriptome pairs. Metagenomic data generation and processing were performed at the Broad Institute. After standard sequence- and sample-level quality control as described in Lloyd-Price et al. [[1](#_ENREF_1)], species-level taxonomic abundances were inferred for all samples using MetaPhlAn 2 [[2](#_ENREF_2)] and functional profiling was performed by using HUMAnN 2 [[3](#_ENREF_3)]. The resulting data types including metagenome-based taxonomic abundances and pathway abundance profiles for both metagenomics and metatranscriptomics (summarized as structured pathways from MetaCyc [[4](#_ENREF_4)]) were used as inputs for MaAsLin 2 analysis.

Significance testing with shuffled data

In order to quantify whether MaAsLin 2 and other multivariable association methods identified more significant associations than expected by chance (i.e. when all the shared signal between features and metadata are broken), we repeatedly shuffled the metadata sample labels, applied multivariable association methods on the randomized data to link features to metadata, and compared the number of statistically significant associations obtained with these randomized data to the number of statistically significant associations obtained with the original data based on the unadjusted p-values. For a comprehensive comparison of both count and noncount models in this experiment, prior to data shuffling, we multiplied the species-level taxonomic abundances by 5% of the filtered read counts as a “proxy” for the underlying raw sequencing count data. The procedure was repeated 1,000 times to estimate the null distribution of the detection performance in both baseline and longitudinal models (with the exception of Compound Poisson mixed effects model which was repeated 100 times to save computation time). While the baseline model included all time-invariant covariates (age, antibiotic use, IBD diagnosis), the longitudinal model also included subjects as random effects with an additional time-variant fixed effect i.e. IBD dysbiosis state, as stated below.

Statistical analysis of species, DNA pathways, and RNA pathways

For both species and DNA pathways (whole-community and species-stratified), we regressed the log-transformed relative abundances (with half the minimum relative abundance as pseudo count, the default in MaAsLin 2) using the following per-feature linear mixed-effects model:

*feature ~ (intercept) + diagnosis + diagnosis/dysbiosis + antibiotic use + consent age + (1 | recruitment site) + (1 | subject).*

Additionally, we modeled the log-transformed relative abundances of the whole-community and species-stratified RNA pathways (with half the minimum relative abundance per feature as pseudo count) using the similar linear mixed-effects model as before, while additionally adjusting the corresponding DNA pathways abundance as a continuous covariate to filter out the influence from gene copies:

*RNA feature ~ (intercept) + diagnosis + diagnosis/dysbiosis + antibiotic use + consent age + DNA feature + (1 | recruitment site) + (1 | subject)*

That is, in each per-feature multivariable model, recruitment sites and subjects were included as random effects to account for the correlations in the repeated measures (denoted by (1 | recruitment site) and (1 | subject) respectively) and the transformed abundances of each feature was modeled as a function of diagnosis (a categorical variable with non-IBD as the reference group) and dysbiosis state as a nested binary variable (with non-dysbiotic as reference) within each IBD phenotype (UC, CD, and non-IBD), while adjusting for consent age as a continuous covariate, and antibiotics as a binary covariate. Nominal p-values were adjusted for multiple hypothesis testing with a target false discovery rate of 0.25 with this FDR chosen to match the original study.

**References**

1. Lloyd-Price J, Arze C, Ananthakrishnan AN, Schirmer M, Avila-Pacheco J, Poon TW, et al. Multi-omics of the gut microbial ecosystem in inflammatory bowel diseases. Nature. 2019;569(7758):655-62. Epub 2019/05/31. doi: 10.1038/s41586-019-1237-9. PubMed PMID: 31142855; PubMed Central PMCID: PMC6650278.

2. Truong DT, Franzosa EA, Tickle TL, Scholz M, Weingart G, Pasolli E, et al. MetaPhlAn2 for enhanced metagenomic taxonomic profiling. Nat Methods. 2015;12(10):902-3. Epub 2015/09/30. doi: 10.1038/nmeth.3589. PubMed PMID: 26418763.

3. Franzosa EA, McIver LJ, Rahnavard G, Thompson LR, Schirmer M, Weingart G, et al. Species-level functional profiling of metagenomes and metatranscriptomes. Nat Methods. 2018;15(11):962-8. Epub 2018/11/01. doi: 10.1038/s41592-018-0176-y. PubMed PMID: 30377376; PubMed Central PMCID: PMC6235447.

4. Caspi R, Altman T, Billington R, Dreher K, Foerster H, Fulcher CA, et al. The MetaCyc database of metabolic pathways and enzymes and the BioCyc collection of Pathway/Genome Databases. Nucleic Acids Res. 2014;42(Database issue):D459-71. Epub 2013/11/15. doi: 10.1093/nar/gkt1103. PubMed PMID: 24225315; PubMed Central PMCID: PMC3964957.
