# Supplementary figures and images for: Multivariable association discovery in population-scale meta-omics studies
Source: PLoS Comput Biol. 2021 Nov 16;17(11):e1009442. doi: 10.1371/journal.pcbi.1009442 (PMC8714082; doi:10.1371/journal.pcbi.1009442)

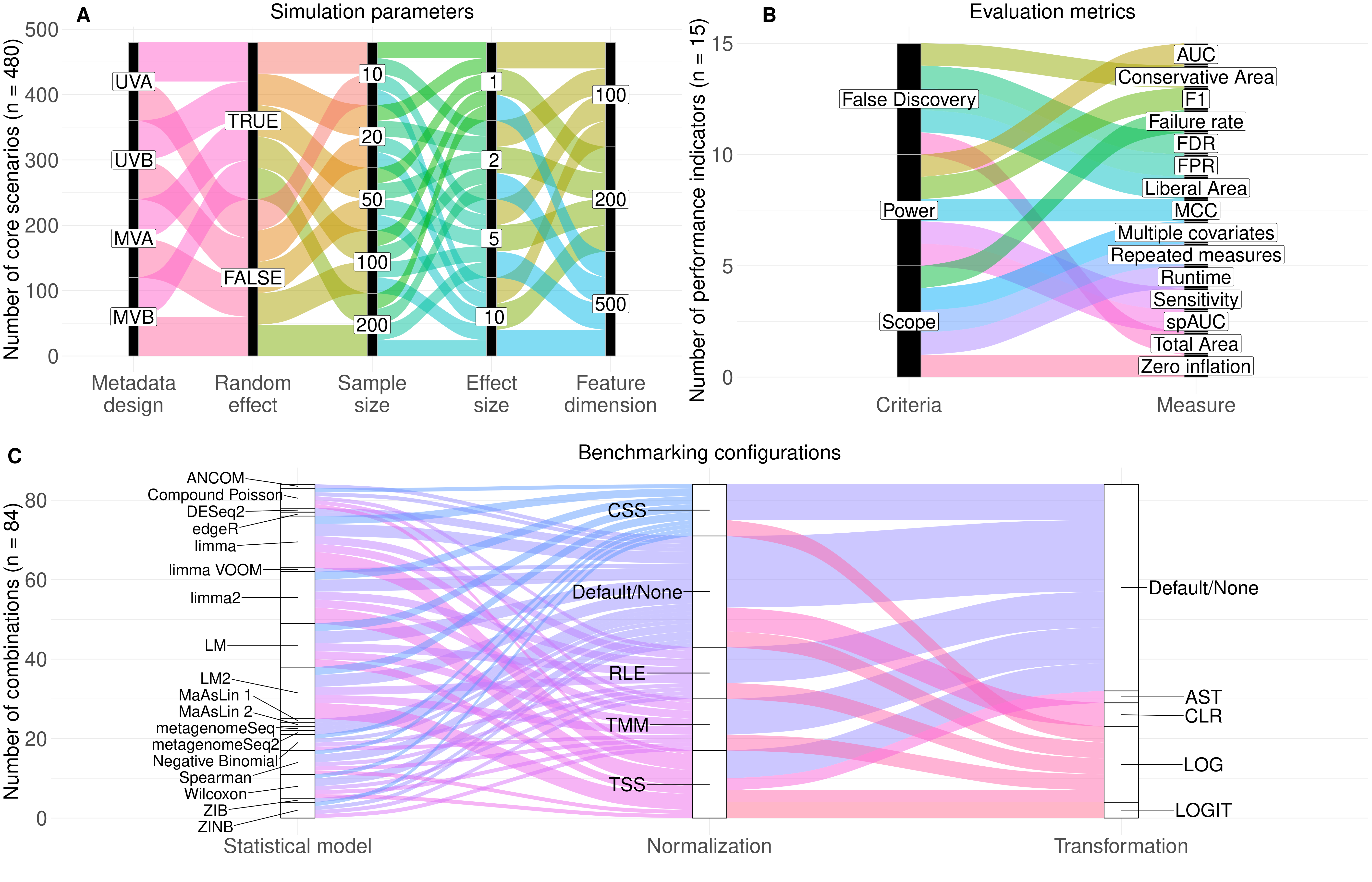

Supplement: S1 Fig — A) Four broad metadata designs commonly encountered in microbiome epidemiology for varying sample size, effect size, and feature dimensions are considered: UVA (Single continuous metadata), UVB (Single binary metadata), MVA (Multiple independent metadata), and MVB (Multiple correlated metadata). For each of this broad metadata design, both cross-sectional and longitudinal cases are evaluated (S1 Text). B) Three aspects of performance are considered: (i) false discovery, (ii) sensitivity, and (iii) scope and computational efficiency of the associated software, each comprising multiple evaluation metrics (S2 Text). C) A combination of statistical models, normalization, and transformation schemes are employed to the synthetic datasets for a variety of association methods, leading up to 84 combinations of normalization/transformation, zero-inflation, and regression models. (TIFF) [file pcbi.1009442.s018.tiff]

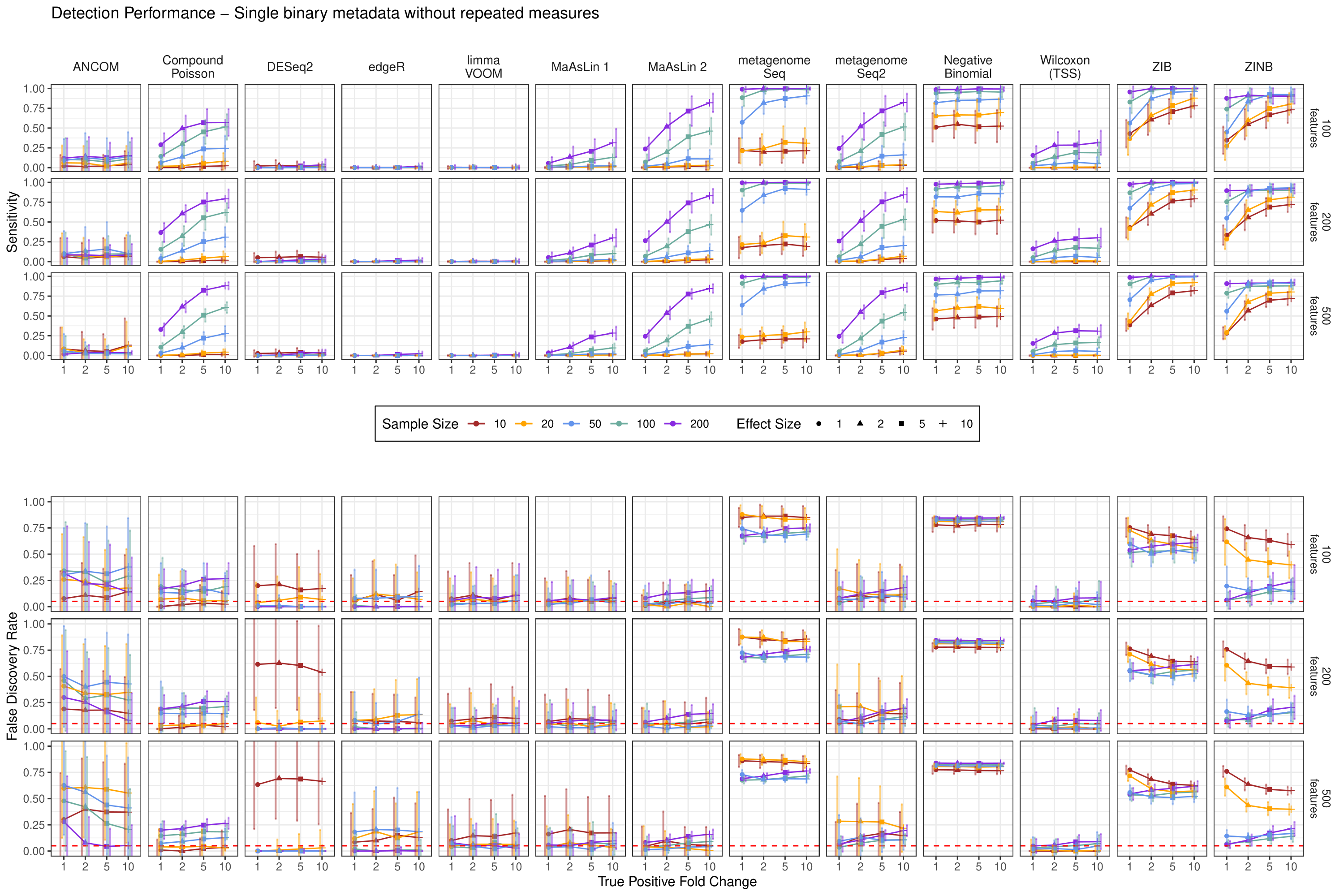

Supplement: S2 Fig — Both sensitivity and false discovery rates (FDR) are shown for the best-performing methods from each class of methods (as measured by average F1 score). Values are averages over 100 iterations for each parameter combination. The x-axis (effect size) within each panel represents the linear effect size parameter; a higher effect size represents a stronger association. For visualization purposes, only the best-performing methods from each class of models (as measured by average F1 score) are shown. Red line parallel to the x-axis is the target threshold for FDR in multiple testing. Methods are sorted by increasing order of average F1 score across all simulation parameters in this setting. All methods were parallelized using custom bash scripts in a high-performance computing environment and methods unable to process specific simulation configurations due to high computational overhead or slow convergence were omitted for those cases. (TIFF) [file pcbi.1009442.s019.tiff]

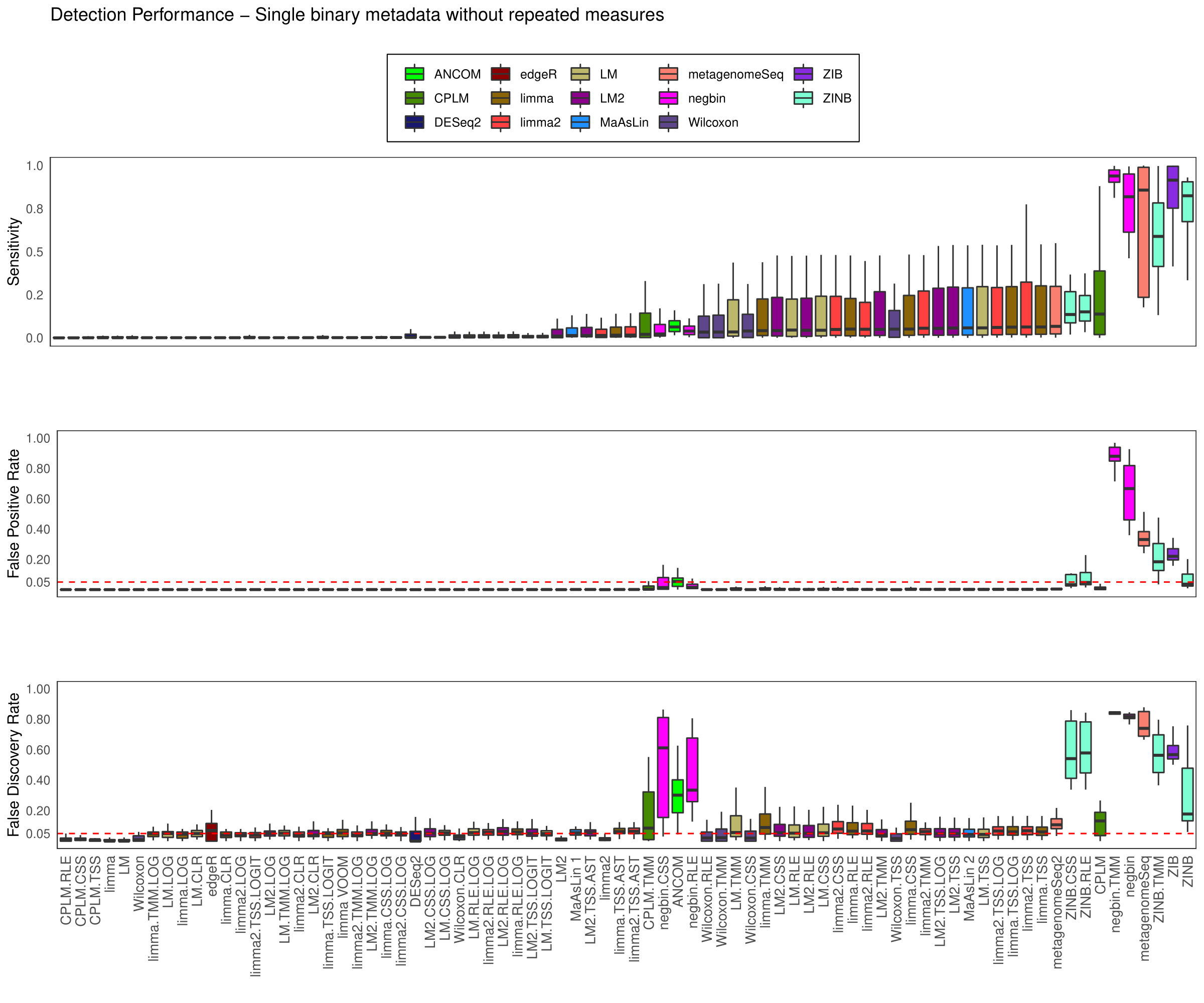

Supplement: S3 Fig — Detection performance measures (Sensitivity, FPR, FDR) for all methods are provided. Values are averages over all parameter combinations each summarized over 100 iterations. Red line parallel to the x-axis is the target threshold for FDR in multiple testing. Methods are sorted by increasing order of average F1 score across all simulation parameters in this setting. (TIFF) [file pcbi.1009442.s020.tiff]

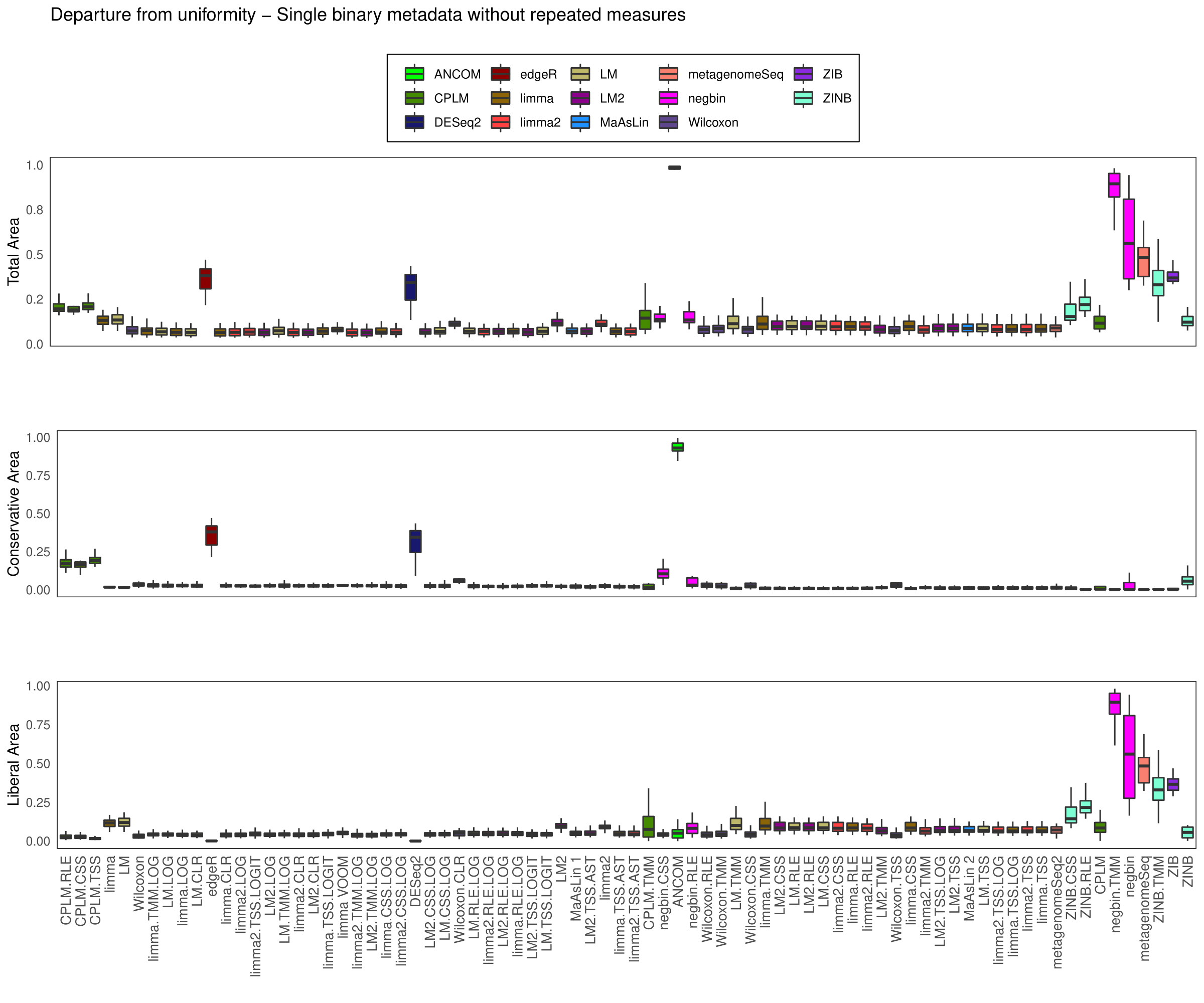

Supplement: S4 Fig — P-value calibration measures as measured by ‘departure from uniformity’ (Liberal Area, Conservative Area, Total Area; S2 Text) for all methods are displayed. Values are averages over all parameter combinations each summarized over 100 iterations. Red line parallel to the x-axis is the target threshold for FDR in multiple testing. Methods are sorted by increasing order of average F1 score across all simulation parameters in this setting. (TIFF) [file pcbi.1009442.s021.tiff]

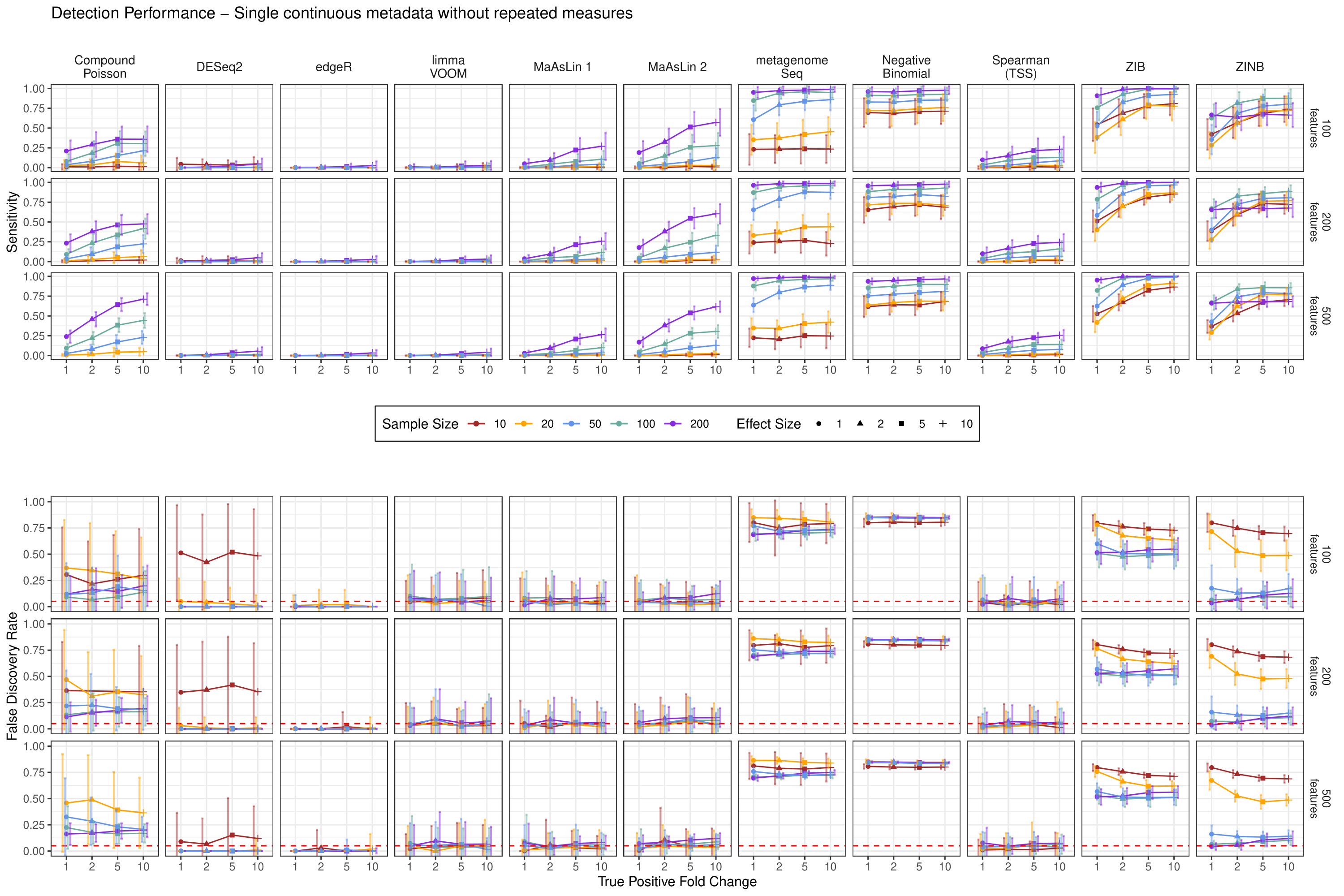

Supplement: S5 Fig — Both sensitivity and false discovery rates (FDR) are shown for the best-performing methods from each class of methods (as measured by average F1 score). Values are averages over 100 iterations for each parameter combination. The x-axis (effect size) within each panel represents the linear effect size parameter; a higher effect size represents a stronger association. For visualization purposes, only the best-performing methods from each class of models (as measured by average F1 score) are shown. Red line parallel to the x-axis is the target threshold for FDR in multiple testing. Methods are sorted by increasing order of average F1 score across all simulation parameters in this setting. All methods were parallelized using custom bash scripts in a high-performance computing environment and methods unable to process specific simulation configurations due to high computational overhead or slow convergence were omitted for those cases. (TIFF) [file pcbi.1009442.s022.tiff]

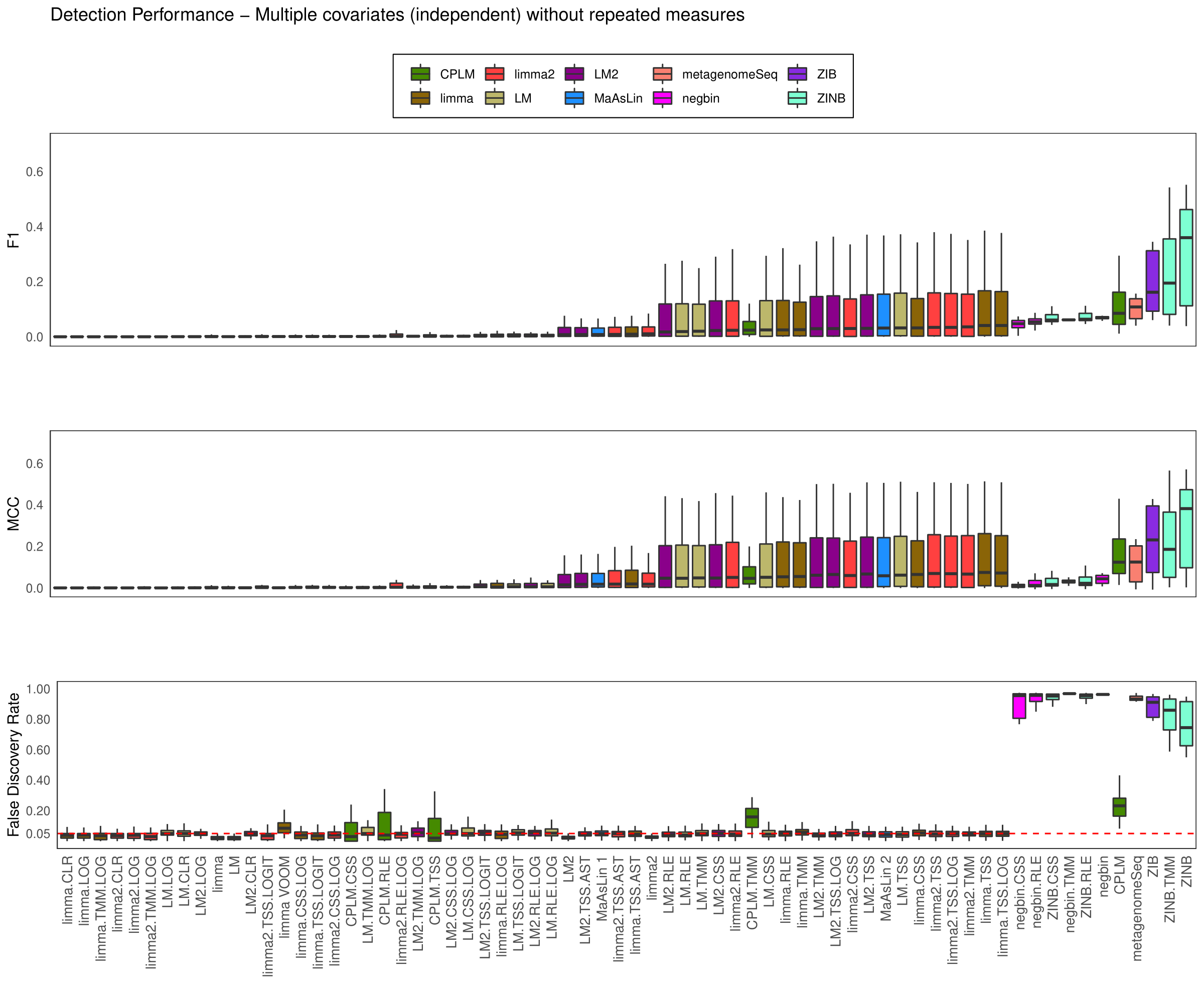

Supplement: S6 Fig — Detection performance measures (F1 score, Matthew’s correlation coefficient, FDR) for all methods are displayed. Values are averages over all parameter combinations each summarized over 100 iterations. Red line parallel to the x-axis is the target threshold for FDR in multiple testing. Methods are sorted by increasing order of average F1 score across all simulation parameters in this setting. (TIFF) [file pcbi.1009442.s023.tiff]

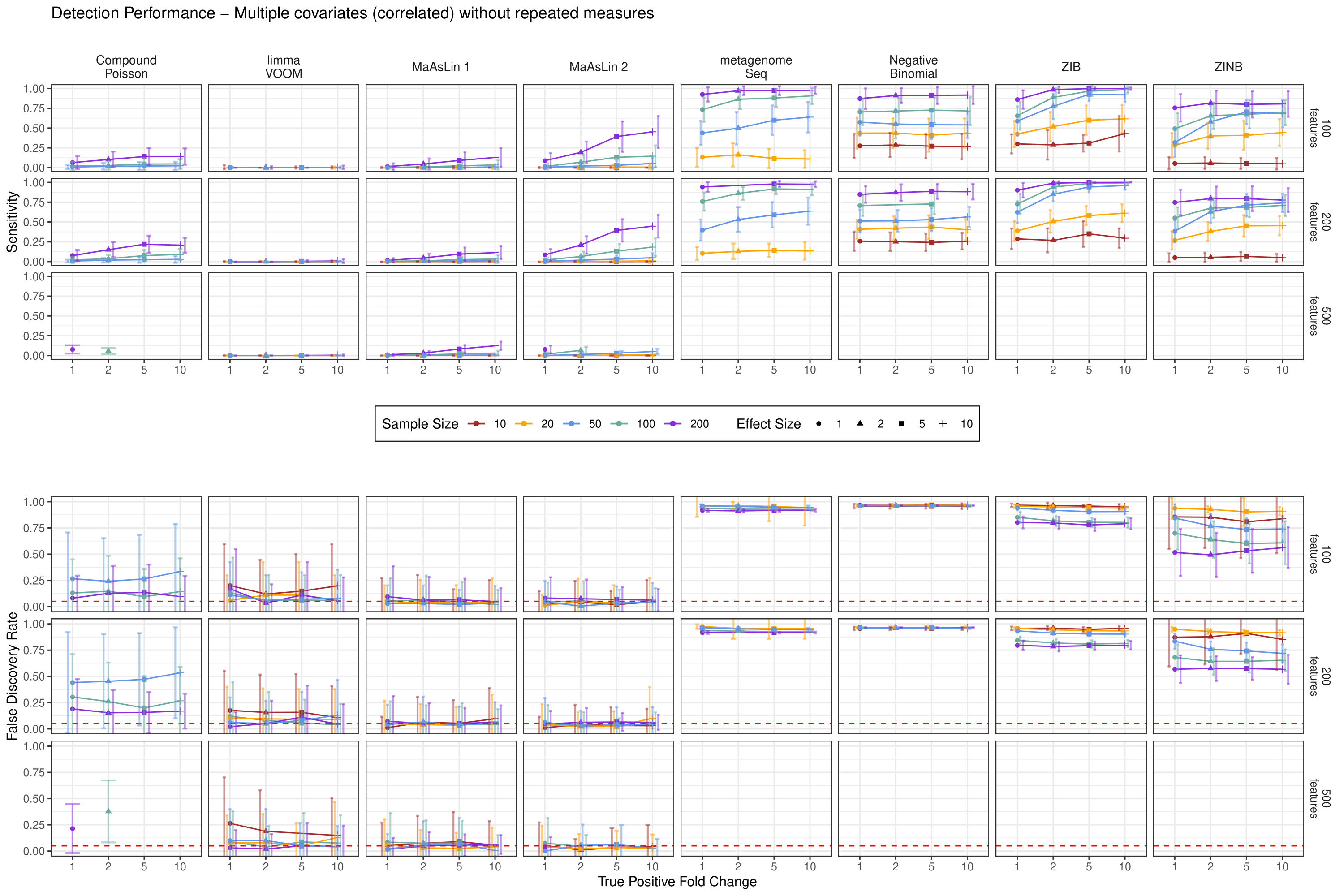

Supplement: S7 Fig — Both sensitivity and false discovery rates (FDR) are shown for the best-performing methods from each class of methods (as measured by average F1 score). Values are averages over 100 iterations for each parameter combination. The x-axis (effect size) within each panel represents the linear effect size parameter; a higher effect size represents a stronger association. For visualization purposes, only the best-performing methods from each class of models (as measured by average F1 score) are shown. Red line parallel to the x-axis is the target threshold for FDR in multiple testing. Methods are sorted by increasing order of average F1 score across all simulation parameters in this setting. All methods were parallelized using custom bash scripts in a high-performance computing environment and methods unable to process specific simulation configurations due to high computational overhead or slow convergence were omitted for those cases. (TIFF) [file pcbi.1009442.s024.tiff]

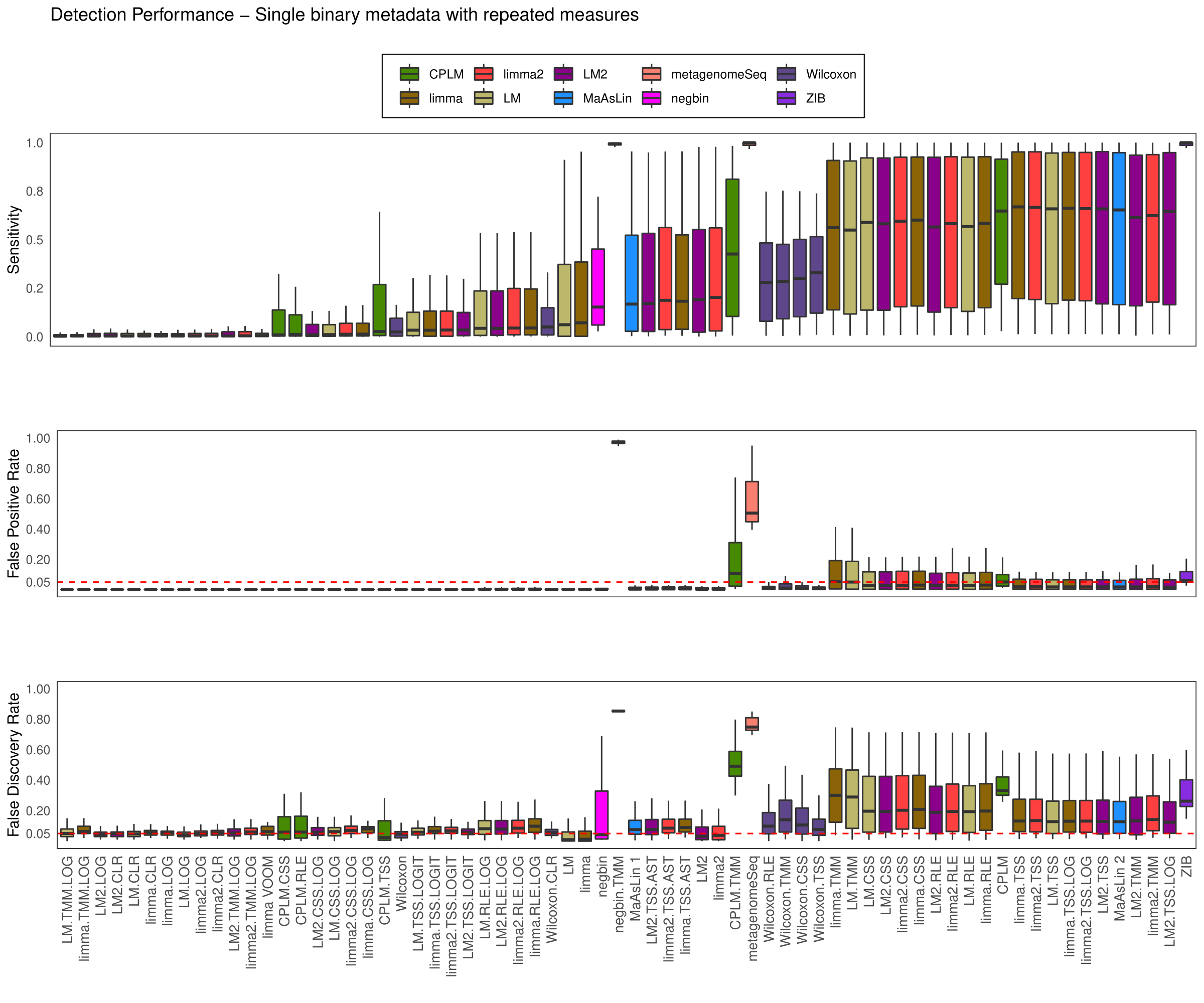

Supplement: S8 Fig — Detection performance measures (Sensitivity, FPR, FDR) for all methods are displayed. Values are averages over all parameter combinations each summarized over 100 iterations. Red line parallel to the x-axis is the target threshold for FDR in multiple testing. Methods are sorted by increasing order of average F1 score across all simulation parameters in this setting. (TIFF) [file pcbi.1009442.s025.tiff]

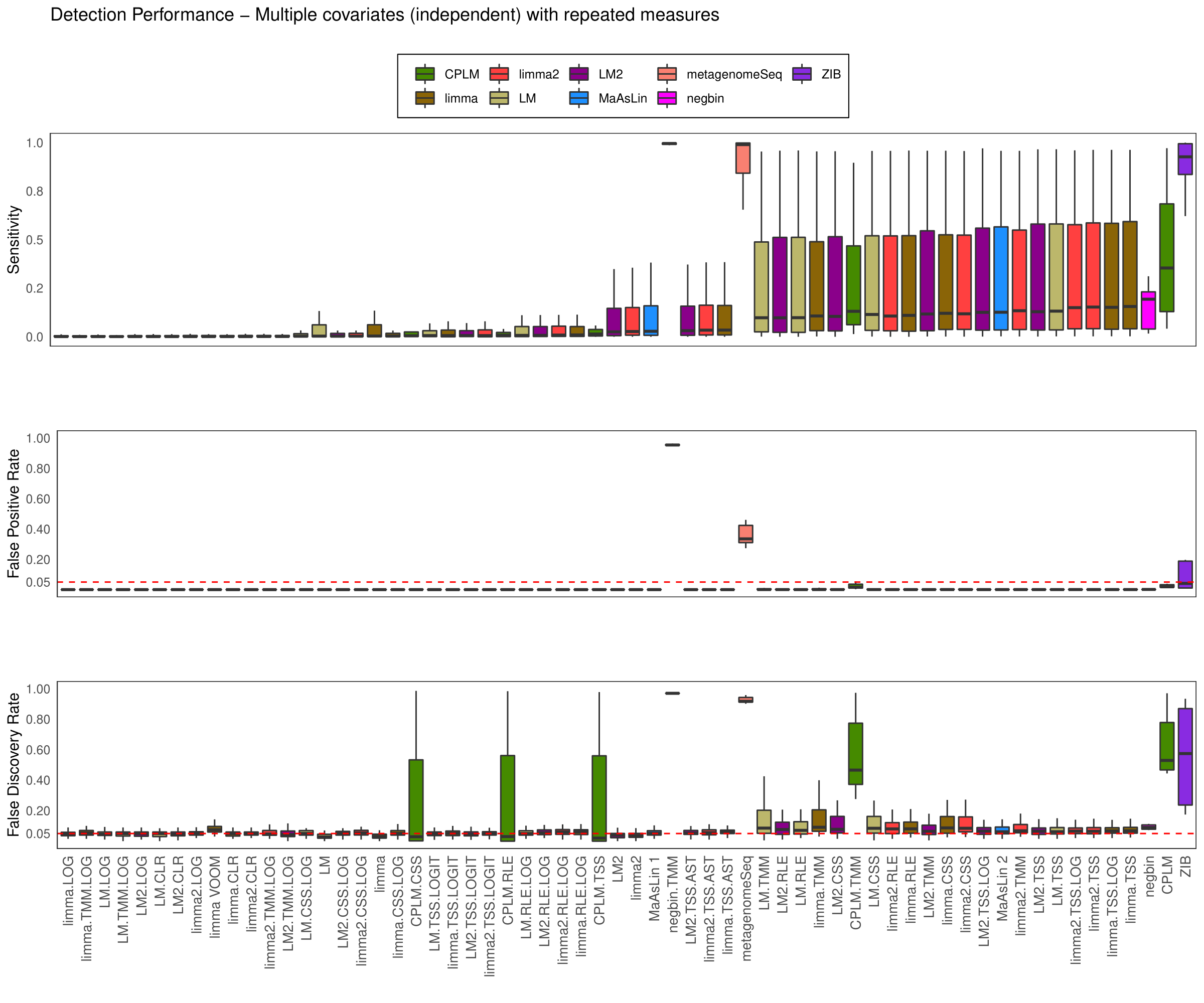

Supplement: S9 Fig — Detection performance measures (Sensitivity, FPR, FDR) for all methods are displayed. Values are averages over all parameter combinations each summarized over 100 iterations. Red line parallel to the x-axis is the target threshold for FDR in multiple testing. Methods are sorted by increasing order of average F1 score across all simulation parameters in this setting. (TIFF) [file pcbi.1009442.s026.tiff]

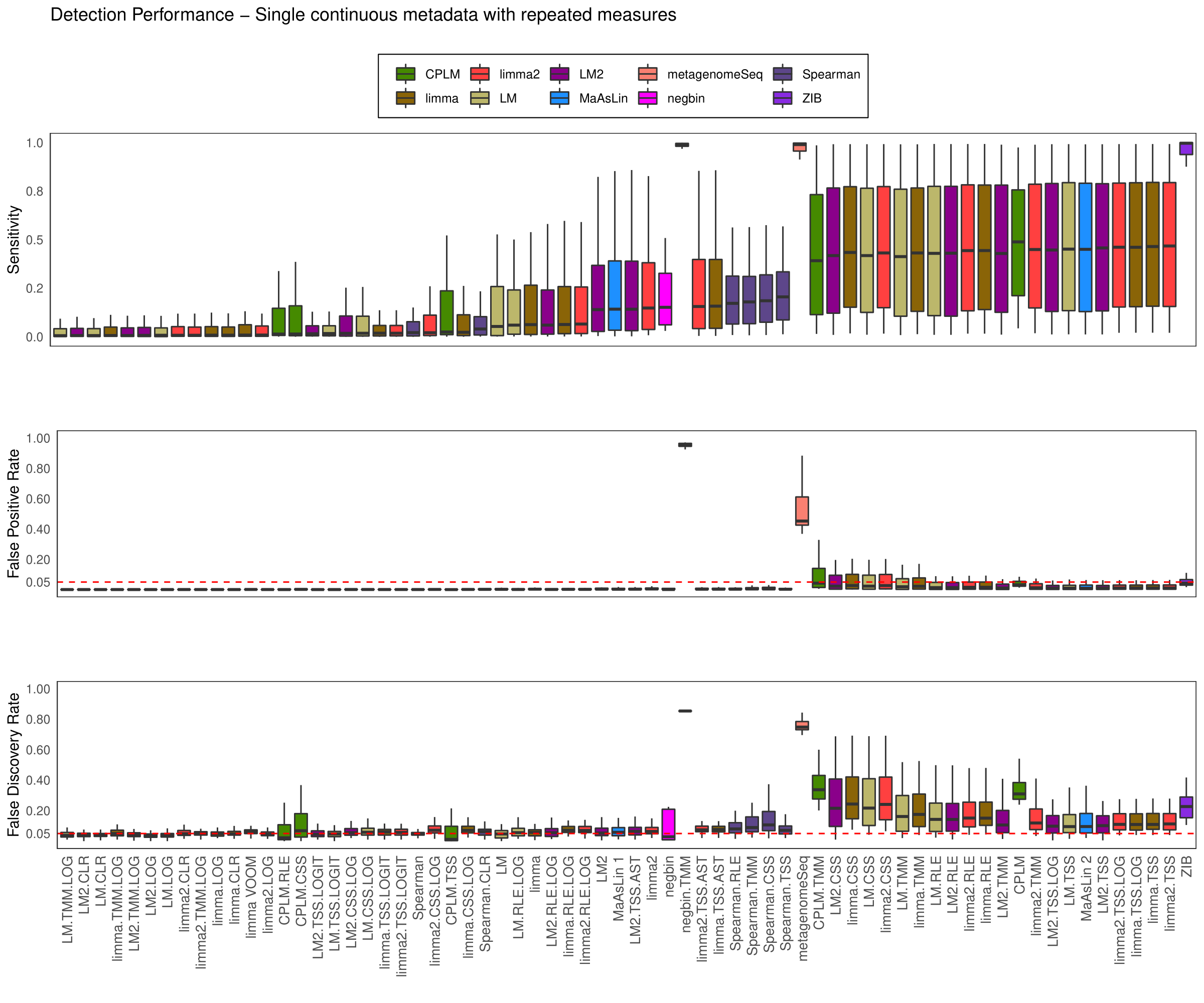

Supplement: S10 Fig — Detection performance measures (Sensitivity, FPR, FDR) for all methods are displayed. Values are averages over all parameter combinations each summarized over 100 iterations. Red line parallel to the x-axis is the target threshold for FDR in multiple testing. Methods are sorted by increasing order of average F1 score across all simulation parameters in this setting. (TIFF) [file pcbi.1009442.s027.tiff]

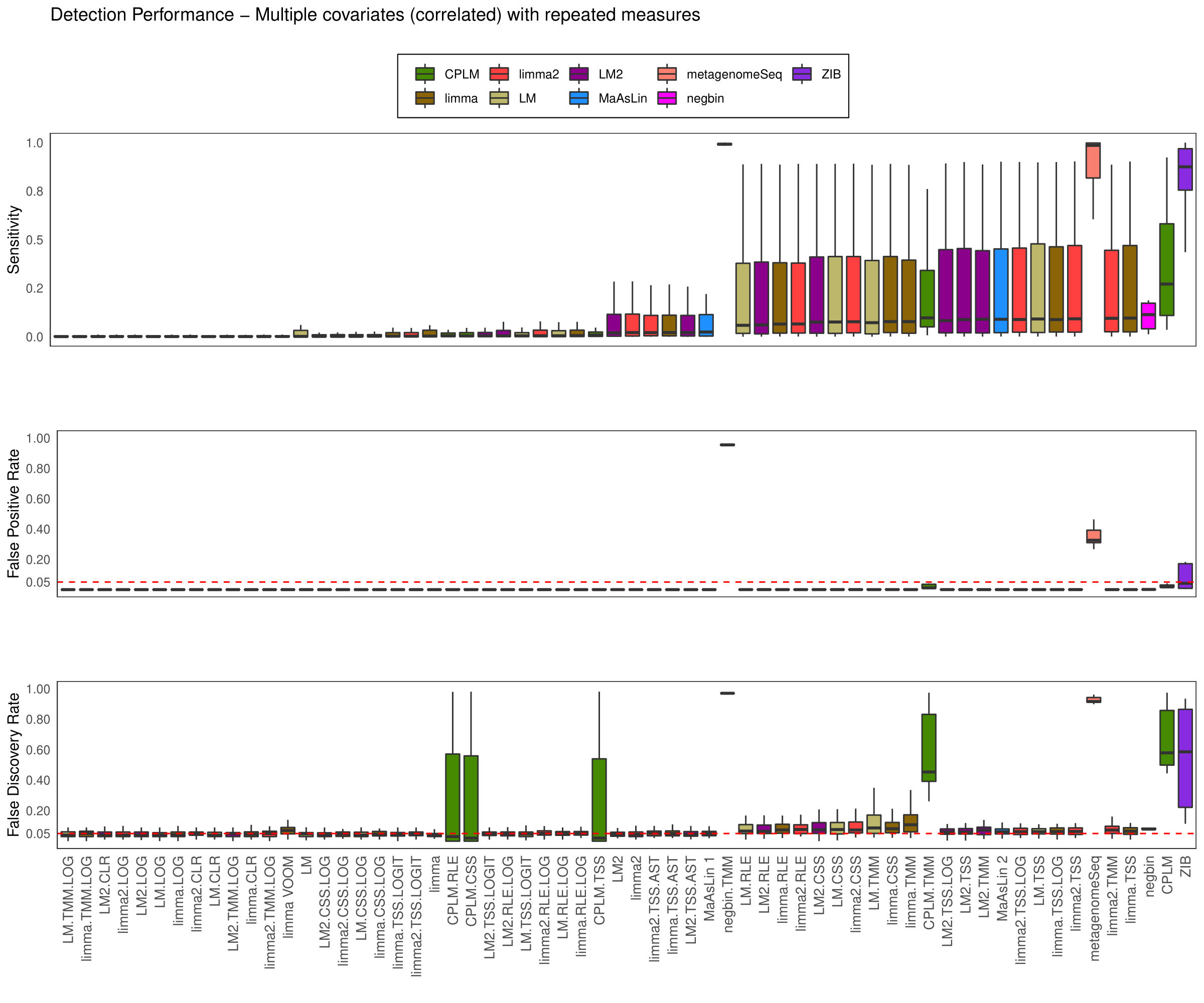

Supplement: S11 Fig — Detection performance measures (Sensitivity, FPR, FDR) for all methods are displayed. Values are averages over all parameter combinations each summarized over 100 iterations. Red line parallel to the x-axis is the target threshold for FDR in multiple testing. Methods are sorted by increasing order of average F1 score across all simulation parameters in this setting. (TIFF) [file pcbi.1009442.s028.tiff]

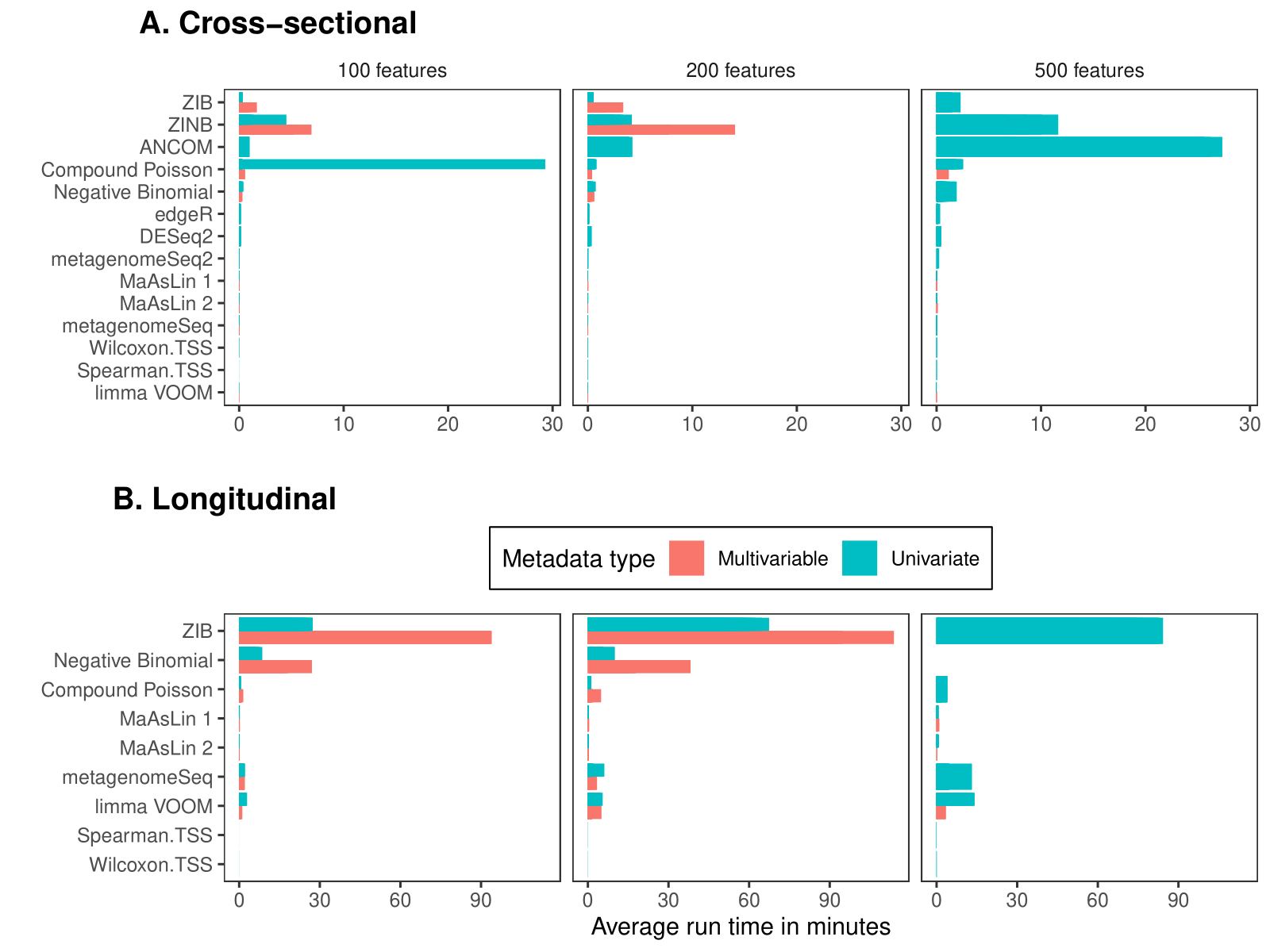

Supplement: S12 Fig — CPU time (in minutes) is shown for all models faceted by feature dimension (100, 200, 500) and colored by metadata design (i.e., univariate and multivariable) in both cross-sectional (top) and longitudinal (bottom) settings. Values are averages over 100 iterations for each parameter combination. All methods were parallelized using custom bash scripts in a high-performance computing environment and methods unable to process specific simulation configurations due to high computational overhead or slow convergence were omitted for those cases. (TIFF) [file pcbi.1009442.s029.tiff]

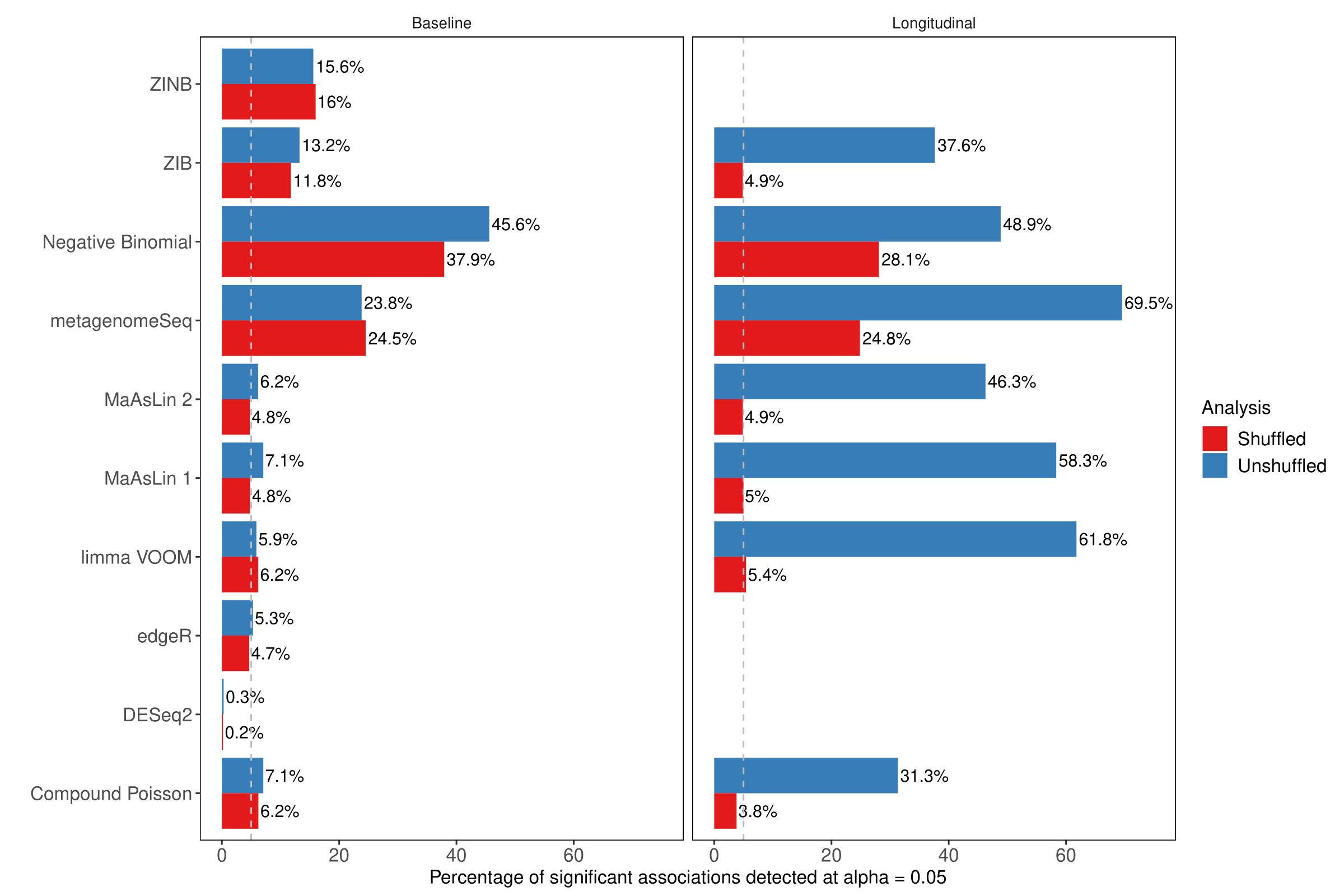

Supplement: S13 Fig — MaAsLin 2’s default linear model produced a consistently lower proportion of significant associations on negative training data (or repeatedly shuffled training set) (averaged over 1,000 permutations) than the positive training (unshuffled) counterpart in both baseline and longitudinal models (S3 Text). Values are average percentages of statistically significant associations (unadjusted P < 0.05) summarized over 1000 permutations. Dashed line parallel to the y-axis is the desired 5% significance threshold. (TIFF) [file pcbi.1009442.s030.tiff]

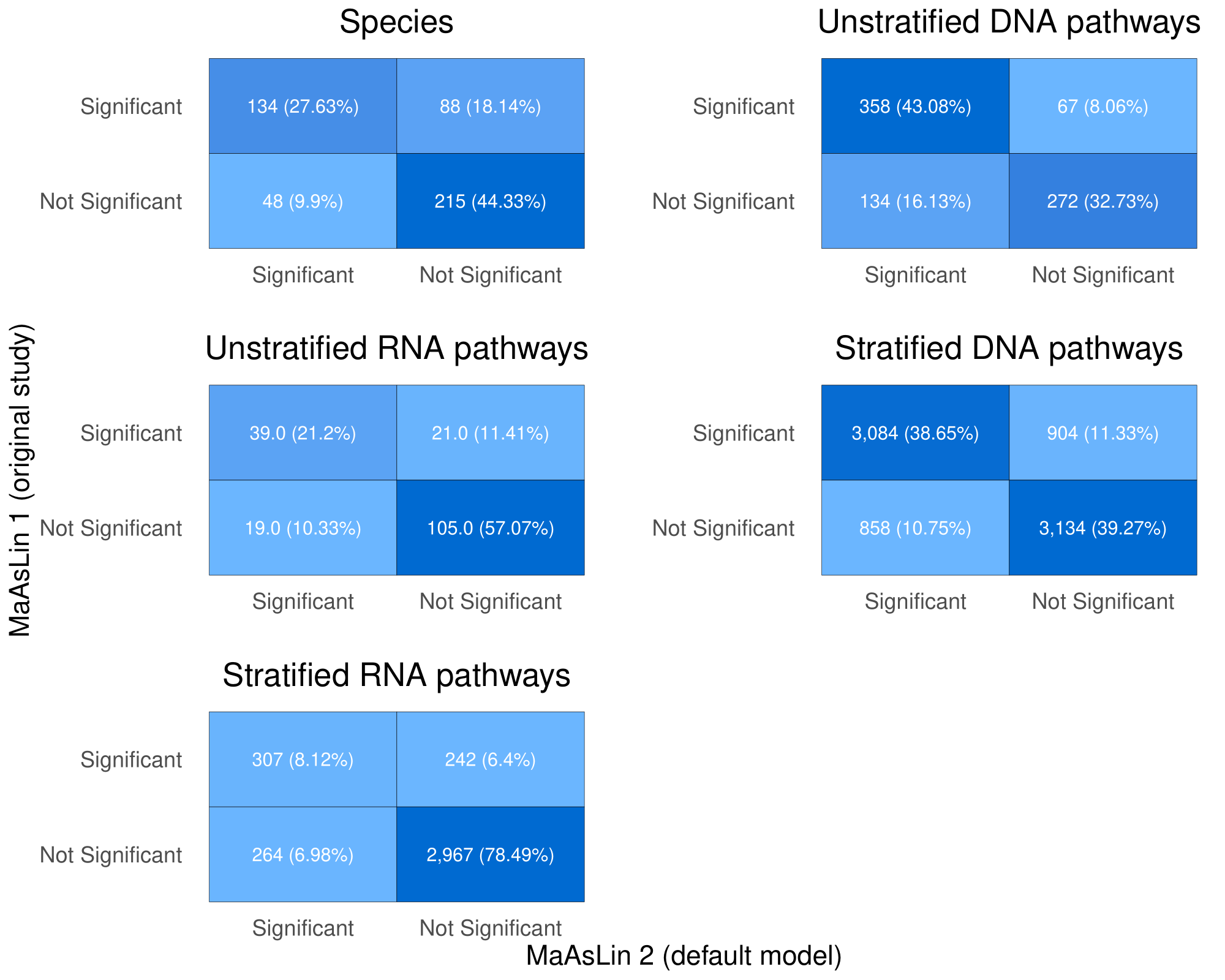

Supplement: S14 Fig — Contingency tables describing the intersection of detected features (across all covariates, restricted to common associations found by both methods) between MaAsLin 2 and the original study for various data modalities in the IBDMDB dataset (S3 Text). (TIFF) [file pcbi.1009442.s031.tiff]

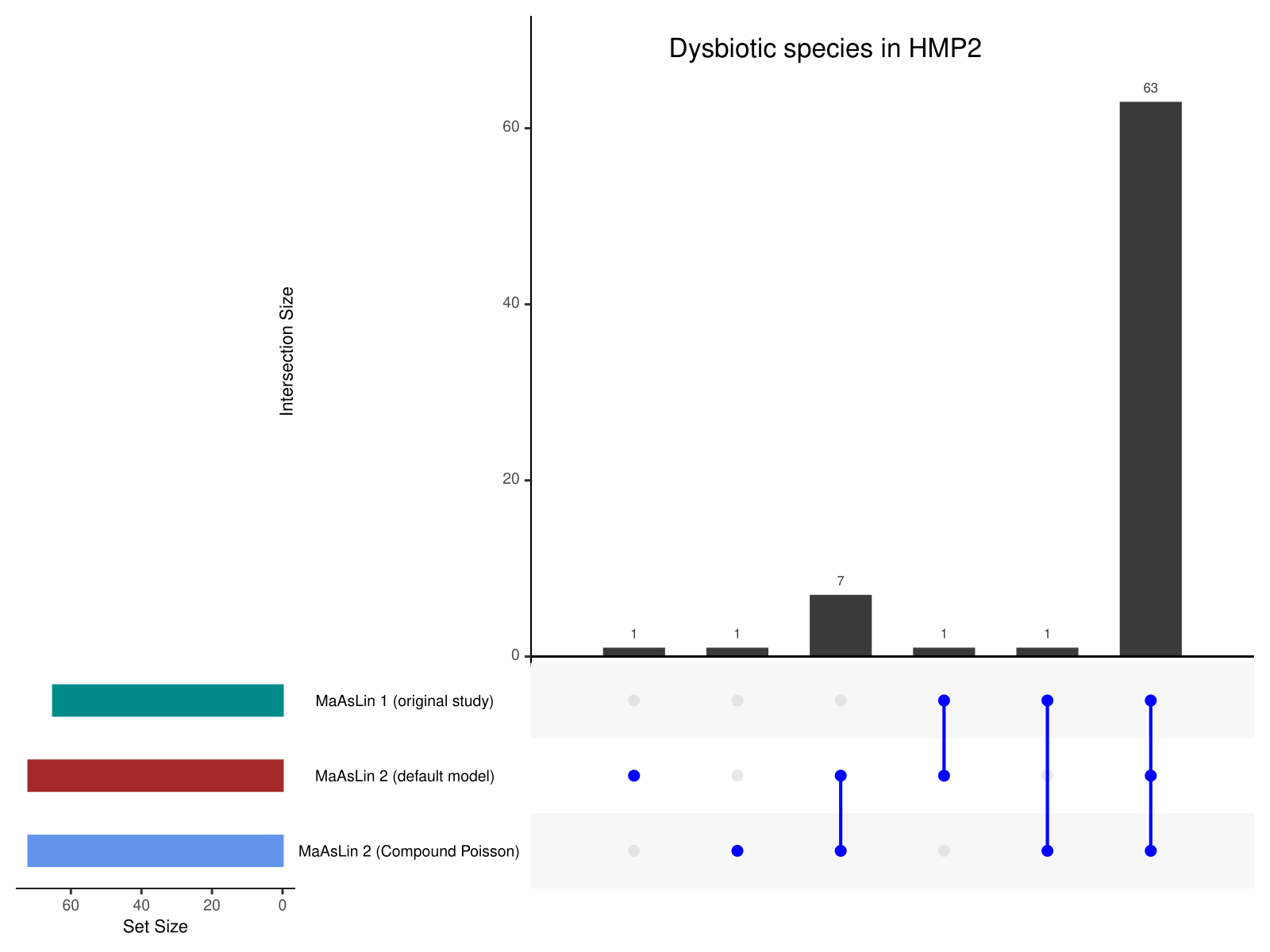

Supplement: S15 Fig — Upset plot describing the intersection of detected dysbiotic taxonomic features between various MaAsLin 2 models in the IBDMDB dataset reveals significant overlap across methods (restricted to common associations found by all methods). A similar pattern was observed for functional profiles (data not shown). (TIFF) [file pcbi.1009442.s032.tiff]
