# Supplementary material for: Multivariable association discovery in population-scale meta-omics studies
Source: PLoS Comput Biol. 2021 Nov 16;17(11):e1009442. doi: 10.1371/journal.pcbi.1009442 (PMC8714082; doi:10.1371/journal.pcbi.1009442)
Supplement: Attachment — Submitted filename: Response_Letter.docx [file pcbi.1009442.s033.pdf]

HARVARD T.H. CHAN SCHOOL OF PUBLIC HEALTH

Curtis Huttenhower
Professor of Computational Biology and Bioinformatics
Department of Biostatistics

Dear Editors,

We would like to thank both you and all three referees for the thoughtful comments and close attention to our work provided during review. We have carefully gone over the comments and responded to each individually below. The most important are:

- While Reviewer 3 may have overlooked several elements that were already included in the manuscript, which we have expanded below, we appreciate the enthusiasm expressed by Reviewers 1 and 2, who deemed the work '<u>an invaluable addition to the current list of toolsets available to microbiome/metagenome researchers</u>' and '<u>informative, well-executed, and clearly written</u>', respectively.

- In agreement with Reviewer 2, we clarified the novelty of MaAsLin 2 as one of the <u>first methods in the field</u> that preserves statistical power in the presence of repeated measures and multiple covariates, while accounting for the nuances of meta-omics features and controlling false discovery, unlike many other frequently used testing procedures (**Response Table 1**).

- We further clarified that the benchmarking leading up to this methodology independently provides a more comprehensive evaluation of association methods for microbiome epidemiology compared to prior work, through a <u>comprehensive evaluation of critical aspects not considered by previous studies</u> (e.g., multiple covariates and repeated measures, among others; **Response Table 2**).

- Finally, we point out that, as potentially misunderstood by the reviewers, there is no previous MaAsLin 1 publication. Instead, the current version represents a software update, and this is the first manuscript associated with the methodology, despite its wide use in practice. Of note, it was used as part of the previously published HMP2 study in Nature, and its open-source Bioconductor package has already been downloaded more than 8,000 times prior to independent publication (**Response Table 3**).

Thus, we hope to emphasize that 1) <u>no previous method exists</u> to address the problems solved by MaAsLin 2, 2) the evaluations we carried out during its development <u>substantially exceed any that were carried out previously</u>, and 3) there is a widespread, <u>unmet need in the field of applied microbiome epidemiology</u> for such a method. Our point-by-point response to these issues is summarized below. We attach two versions of the updated manuscript, one with modified sections highlighted, and a version without markup; we appreciate the opportunity to make these clarifications, and we are open to any further discussion or suggestions.

Sincerely,

Curtis Huttenhower
Professor of Computational Biology and Bioinformatics
Harvard T. H. Chan School of Public Health

Department of Biostatistics
655 Huntington Ave
Building 1, Room 413
Boston, MA 02115

TEL 617 432 4912
FAX 617 432 5619
chuttenh@hsph.harvard.edu

Referee #1

> **The paper addresses a need in the field for robust statistical tools to study associations between metagenome and covariates such as location, environment, phenotypes, diet, health outcome and more. While there are many existing association methods, most methods focus on univariate associations for ease of interpretability. MaAsLin 2 is one of several methods that not only test for multivariate associations, it also provides multiple statistical models, normalization and transformation schemes in a single R package. I believe it'll be an invaluable addition to the current list of toolsets available to microbiome/metagenome researchers.**

Response 1.1 We thank the reviewer for the summary of our work, especially for their positive comments on the originality of this work and appreciation on the importance of the associated software.

> **The authors created a comprehensive benchmarking framework that uses synthetic abundances generated by SparseDOSSA. It is in my opinion a suitable alternative to simulating sequencing reads if the purpose is to evaluate the power of the differential abundance analysis tools. In the absence of an experimentally-validated gold standard, a synthetic abundance dataset might be the next best thing for a systematic controlled evaluation of statistical performance. That said, I'm curious if the biases and errors introduced by taxonomic and functional classifiers might affect the performance of these tools. The authors used a variety of widely accepted metric such as FDR, AUC and F1 score to pit the performance of MaAsLin 2 against other covariate association software and was able to show convincingly that MaAsLin 2 does indeed improve sensitivity in a variety of synthetic datasets while controlling for FDR.**

Response 1.2 Many thanks to Reviewer 1 for their supportive feedback about this study, especially the need for a systematic controlled evaluation of statistical performance in the absence of an experimentally validated 'gold standard'. If we understand the question associated with this comment correctly, it would be whether issues introduced during the process of quantifying features from sequencing reads "matters" or should be evaluated in this context. That is, there is always some noise introduced in taxonomic profiles or gene profiles when computed from shotgun metagenomic sequences. If this is what's meant by the question, then our thinking would be 1) yes, in principle these could affect different models differently (although they should usually be affected identically), but 2) it's not clear that it's the models' responsibilities to fix this (as opposed to that of the profilers or another preprocessing / normalization step).

As a simple example, consider between-run batch effects, a particularly extreme case of bias and error introduced by upstream sequence processing. It can certainly affect differential abundance model results if batches are confounded with biological variables of interest. It could in principle also affect different hypothesis tests differently; for example, a model that's less sensitive to global block differences (and maybe more sensitive to individual feature differences) might have more false positives as a result. However, we would argue that such biases or errors should not necessarily be resolved as part of a modeling framework such as MaAsLin, but either during the profiling process itself, or "in between" as a prior normalization. As examples, we have a preprocessing model for such effects in review (https://doi.org/10.1101/2020.08.31.261214), as does Michael Wu's lab (https://ww2.amstat.org/meetings/jsm/2021/onlineprogram/AbstractDetails.cfm?abstractid=315506).

To reflect this point, we have added the following text to this effect in the Discussion (**Page 28**):

'Third, it is not possible to capture the full range of differential biases and errors introduced by various bioinformatics pipelines using a single, representative template dataset, as considered here. To this end, multiple, diverse taxonomic and functional template datasets can be considered for future benchmarking, potentially in combination with other upstream simulation frameworks such as CAMISIM[48] to investigate the effect of sequence assembly, genome binning, batch effects, taxonomic binning, taxonomic profiling, and other steps on differential analysis performance.'

Here are some comments:

1. **Is there a difference between the standardization procedure of SparseDOSSA vs rarefying?**

Response 1.3 The standardization procedure of SparseDOSSA is carried out during spike-in to ensure that the counts of the modified features are not dominated by the values of the target metadata but rather distributed similarly to real data. That is, it "scales" data-metadata relationships so that they are essentially independent of covariates' unit of measurement. This is separate from a rarefaction procedure, which would provide an alternative to data normalization to account for library size differences. In other words, rarefaction is one step that can help ensure meaningful comparisons between samples, regardless of features' association with the metadata, whereas the standardization procedure of SparseDOSSA ensures that the spiked-in features are not influenced by the range of values of the target metadata to enable meaningful head-to-head comparisons of various differential analysis methods.

2. **From the attached manuscript, SparseDOSSA feature-metadata spike in looks like its magnitude is a linear function of the selected metadata. Is it able to simulate non-linear associations?**

Response 1.4 In agreement, the default SparseDOSSA spike in procedure induces an effect size as a linear function of the selected metadata. However, the metadata matrix is a user-defined parameter which can be modified before analysis to contain nonlinear input variables (e.g., smoothing splines) to induce non-linear associations. We have occasionally used it this way (e.g., to introduce interaction effects), although not in this study. We acknowledge that this constitutes an important future direction which we have now added in the **Discussion** section (**Page 28**) as follows:

'Fourth, while we have focused on linear associations in this study, non-linear associations may also be of interest (as in other types of molecular epidemiology).'

3. **As a follow up to point 2, is the reason for the good FDR performance of linear models a testament to the strength of linear models in general or a result of the way feature spike ins are generated?**

Response 1.5 We agree that, in this setting, the FDR control of linear models is likely an effect of both (i) the strength of these models in controlling the FDR in general and (ii) the linear spike-in associations induced by SparseDOSSA. However, this is also true for essentially all of the other models evaluated as well: negative Binomial, ANCOM, metagenomeSeq, and others also primarily describe linear relationships. Additionally, the accuracy of MaAsLin / linear models is also retained in the second set of validations we did using the HMP2 dataset (as described in the **Significance testing with shuffled data** subsection in **Methods**), which is independent from the synthetic evaluation based on SparseDOSSA (and of course not guaranteed to be linear). In particular, we ruled out the effect of false positive findings using a rigorous permutation-based approach by randomly shuffling the dataset many times, which served as a justification for applying LM and other related models that consistently maintained the false positive rates below the desired 5% significance threshold, which is not true for other metagenomics-

focused methods such as metagenomeSeq or count models such as Negative Binomial (**Supplementary Fig. 13)**. Since the shuffled versions of the same "real" dataset can be considered a negative 'gold standard' (unaffected by the linearity assumption), we conclude that, in general, FDR control is surprisingly robust for linear models.

4. The use of the word 'feature' to describe microbial abundance can becoming confusing at times. Nevertheless, I don't see a good way around it except to refrain from using features to describe metadata where possible.

Response 1.6 We agree, and we have carefully gone over the manuscript to ensure that we only use the term 'feature' for microbial taxonomic or functional features, and not to describe metadata. Although the term is certainly ambiguous in other fields (e.g., machine learning), it should now be consistent at least throughout the manuscript.

5. 'Red flags' were mentioned a few times in section "MaAsLin 2 methodology and validation". In particular, "MaAsLin 2 was the only multivariable method tested that controlled FDR with the fewest 'red flags' across scenarios (Fig. 1C). "A concrete description of what these red flags are might be good.

Response 1.7 In agreement, we have now added the following sentence to further clarify the definition of 'red flag' in the manuscript text (**Page 9**):

'For simplicity, we thus abbreviate any extreme departure from a metric's best possible value as a 'red flag'.'

6. As a follow up to point 5, Fig. 1C shows the various metrics "averaged over all simulation parameters". Several models appearing as though they have poor sensitivity despite the supplementary figures showing otherwise. Is that due to averaging across multiple simulation parameter?

Response 1.8 In short, yes: some of the methods (including linear models) exhibit a significant boost in power for larger sample sizes and stronger effect sizes, as evident from the supplementary figures. They thus perform well under some circumstances, but sometimes poorly in others (or vice versa).

7. Is the metric scores for all 84 configurations and 480 core scenarios (and after ignoring incompatible combinations) available as a dataset?

Response 1.9 We unintentionally omitted this before, but we have now curated and provided summary measures for all 84 configurations and 480 core scenarios as data tables in the revised manuscript (**Supplementary Datasets S1-S8).**

8. The labels of the subfigures in Fig. 5 are a,b,c whereas the caption referred to b, c, d.

Response 1.10 We apologize for this error which is now fixed in the updated **Fig. 5** as follows:

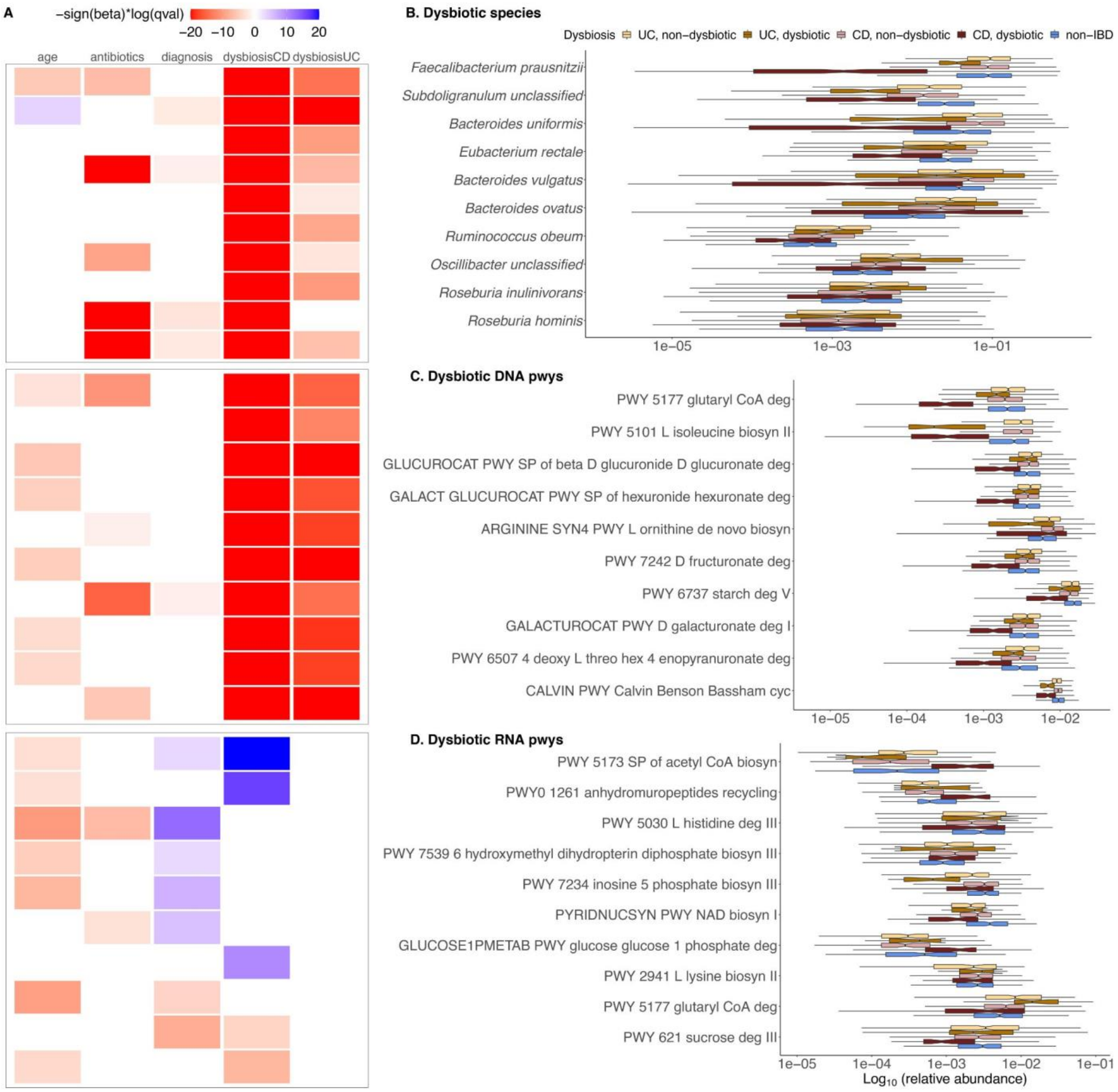

9. Figure caption 1 - "averaged over all simulation parameters (Supplementary Fig. S1B). " should be Supplementary Fig. S1A

Response 1.11 Thanks for noticing this typo which we have now fixed in the manuscript.

10. The labels in Supplementary Figures S3, S4, S6, S8 etc. makes it unwieldy to compare performance between classes of models, normalizations, and transformations.

Response 1.12 We agree that due to a large number of combinations of statistical models, normalizations, and transformations, some of the supplementary figures are unwieldy to compare. However, we did not see much variation within each class of methods to justify an alternative grouping -

and it would be difficult to choose and implement a single convenient standard. As a result, we have attempted to resolve this unwieldiness by displaying only the 'representative' methods in some of the figures (e.g., **Figs. 2-4** and **Supplementary Figures S2, S5, and S7)** as appropriate, retaining the other more detailed versions for the sake of completeness and rigor.

> **11. In Fig. 4, it'll be helpful to arrange the methods so that the order in a) matches b). In fact, if there is some consistency in the arrangement of methods between Fig. 1c, Fig. 2, Fig. 3 and Fig. 4, that'll be great.**

Response 1.13 In an attempt to improve the comparison and interpretation of various methods in **Figs. 2-4**, we have sorted the methods according to the average F1 score. We have further grouped the methods according to their FDR-controlling behavior (e.g., in **Fig. 2**), which clearly conveys the primary conclusion of the paper. We chose this direction over choosing a manual arrangement of methods to ensure a consistent reporting and interpretation within each simulation scenario as well as overall.

Referee #2

Mallick et al. present MaAsLin 2, an updated toolkit for performing multivariable association tests in meta-omics datasets. Overall, I found the paper to be informative, well-executed, and clearly written. I have just a few comments below:

Response 2.1 We thank Reviewer 2 for his or her time, input, and positive evaluation of the manuscript.

I feel the authors could go a bit further in highlighting the improvements of Maaslin2 over Maaslin1. The authors have done a good job in showing improved performance of Maaslin2 over Maaslin1 using an array of carefully simulated datasets. However, figure S13 actually shows the reverse, in which Maaslin1 is more sensitive at the same empirical FPR compared to Maaslin2 when applied to real data (shuffled vs not-shuffled labels). Further, differences in biological results of Maaslin1 and Maaslin2 were not discussed in the final section (448-540). So, there has not been a clear demonstration of the superiority of Maaslin2 over Maaslin1 *in practice*. This is an important point in order to demonstrate a clear advance over the authors' previous work.

Response 2.2 We should first clarify that despite its long use in practice (e.g., PMIDs 23013615, 25887922), the MaAsLin 1 methodology was never previously published. This is certainly nonobvious to readers - and as I'm sure you can imagine, it's been a major item on the lab's to-do list for multiple years. This is thus the first manuscript publishing the method (the "2" version refers to a software reimplementation).

Briefly, the core GLM used in MaAsLin 1 and 2 is the same, but many other features were improved, updated, or replaced, such as the feature selection method; proportional data transformation; inclusion of models for count (in addition to proportional) data; the evaluation of several zero-inflated models; and the entire R/Bioconductor package implementation. MaAsLin 2 in roughly its current, final form was first used in the Integrative Human Microbiome Project (Nature PMID 31142855), and this manuscript represents the first publication of the method and its formal validation as carried out during that study.

To clarify the difference between MaAsLin 1 and 2, we have included a dedicated subsection in the **Methods** section (**Statistical methods**) that specifically calls out these advances and improvements offered by the MaAsLin 2 software (and emphasizes the absence of a previous methodological publication). This is rather long to quote fully in the response, but a relevant subset includes:

- *"Zero-inflated Negative Binomial (ZINB): for fixed effects, we used the zeroinfl() function from the pscl package with the logarithm of library size (for no normalization) or scaling factor (for other normalization schemes such as CSS, RLE, and TMM) as offset. In the absence of a robust random effect implementation of the same, the ZINB method was not considered in the repeated measures settings.*

- *Zero-inflated Beta (ZIB): following Peng et al., we used the gamlss() function from the R package gamlss56 for fixed effects and the ZIBR() function from the ZIBR R package for random effects. In both cases, the features are TSS-normalized before statistical testing.*

- *Compound Poisson (CPLM): we used the cpglm() function from the cplm package and the glmmPQL() function from the MASS package for fixed and random effects respectively. In both cases, we used the logarithm of library size (for no normalization) or scaling factor (for other*

normalization schemes such as CSS, RLE, and TMM) as offset. No offset was used when combined with the TSS-normalized relative counts.

- *MaAsLin 1: we used the default TSS-normalized, arcsine square root-transformed linear model without gradient boosting.*
- *MaAsLin 2: we used the default TSS-normalized, log-transformed linear model with half the minimum relative abundance as pseudo count."*

The analysis presented in Figure S13 is great. I'd appreciate if the authors would add numbers to each bar - it's currently hard to see whether the FPR is above 5% for any of the methods (especially w/o gridlines).

Response 2.3 We thank the reviewer for the comment. In agreement, we have now added percentages to each bar to better showcase the FPR-controlling behavior of various methods (updated **Figure S13**):

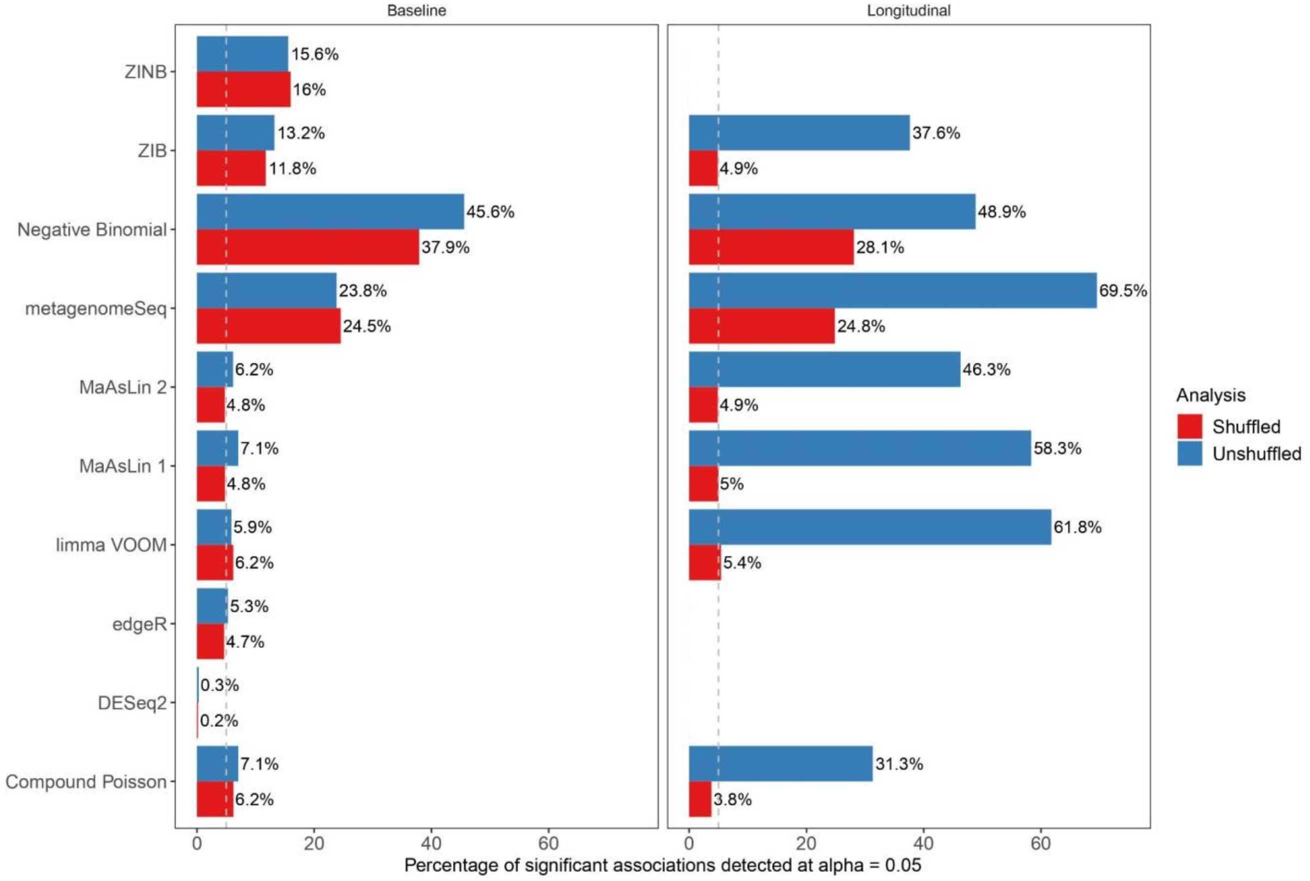

Does Maslin perform mean/variance filtering to improve power (PMID: 20460310)? I do see this mentioned on lines 182, 425, and 746 but not sure if this is built into the software. This could be a really helpful automated step, particularly if the filtering thresholds were chosen using a data driven approach rather than arbitrarily.

Response 2.4. Yes. MaAsLin 2 currently performs (configurable) variance filtering as well as prevalence and abundance filtering as a preprocessing step before model fitting. By default, this entails retaining

only those features that are present (or detected) in at least X (e.g., 10) percent of samples at Y minimum abundance (e.g., 0). If variance filtering is additionally desired, MaAsLin 2 further filters out those features with little or no variance based on a user-defined cutoff Z (e.g., half the median of all variances). In our experience, the optimal filtering threshold (i.e., X, Y, Z) varies from study to study (especially when shifting units between e.g., taxonomic vs. gene vs. pathway features), and as a result, the current implementation lets the user choose an appropriate pre-filter threshold in a data-driven manner. Having said that, we agree that an automated step would further refine the user experience which we intend to do in a future release.

> **For gene based statistical analysis, can the authors comment on whether Maslin2 supports average genome copy number data, which is normalized using the mean abundance across a panel of universally distributed single copy marker genes (PMID: 25885687). Such normalization has also been applied in metatranscriptome analyses (PMID: 20844569). Without this normalization step, differences in genome size can lead to strong associations for microbial housekeeping genes like the ribosome (PMID: 25853934). This is one of the major differences between taxonomic and functional analyses and may be worth discussing in the text.**

Response 2.5 This is an excellent point, and one that we have seen matter in other contexts as well (e.g., 16S rRNA gene copy number normalization for amplicon-based taxonomic profiling). As with other types of "upstream" processing as discussed in **Response 1.2** (e.g., batch correction), we would argue that users should carry out any such normalization prior to differential abundance analysis, rather than during (or after). We agree with the reviewer that this is particularly true since the MaAsLin model is intended to apply to several different types of microbial feature abundances (e.g., MUSiCC-normalized functional KOs or median-normalized metabolites). Since these can be typically done independently as a preprocessing step before running MaAsLin 2, we have avoided the complexity of coupling the two steps and not added them as an option in MaAsLin 2 itself.

Referee #3

There are three main components in this manuscript: (1) extensive simulation studies regarding the sensitivity and false discovery rate of different existing methods for modeling multi-omics data, (2) propose a new method for modeling multi-omics data (which could include taxonomic, metabolomic, etc. data), and (3) apply the proposed method to a modern dataset. I have serious concerns about (1) and (2). Note that the results of (1) are used to justify choices in (2), and the resultant method is then applied in (3).

1. Simulation study: The process of generating simulation data is not described in this paper; a simulation engine called SparseDOSSA is used. Most problematically, the specific data generating process is not described in this manuscript. As a result, key conclusions for this paper have been made based on simulations that cannot be reconstructed or inferred with specificity given the description in the manuscript. SparseDOSSA is software developed by many of the same authors as this manuscript, and is under review as a separate paper (from https://huttenhower.sph.harvard.edu/sparsedossa/ "Citation: Will add once the paper is out"; note this is distinct from https://huttenhower.sph.harvard.edu/maaslin2). At best, it is not possible to understand or interpret the results of the simulation study. Furthermore, the need for a new simulation to investigate the performance of relative abundance methods for modeling microbiome data is unclear -- there are many that already exist (some of which this manuscript cites).

Response 3.1 While we appreciate the reviewer's feedback, we respectfully disagree with the reviewer on the statement that '*key conclusions for this paper have been made based on simulations that cannot be reconstructed or inferred with specificity given the description in the manuscript.*'

The reviewer's assessment that '*The process of generating simulation data is not described in this paper*' is simply not true, as we feel there was significant scientific content in the original manuscript that the reviewer missed. In particular, the reviewer seemed to have overlooked both the SparseDOSSA manuscript (included as part of the submission package) as well as the **Methods** subsection, '**Data for differential feature model evaluations**', where we had multiple, dedicated descriptions of this framework, including '**Synthetic null community abundances**' that detailed the underlying data generation model.

As can be seen from the SparseDOSSA preprint and the associated text in the manuscript, Reviewer 3 may also have missed several aspects of the simulation strategy's novelty not captured by the listed previous methods. In particular, these methods do not accurately reflect real-world microbial feature distributions, influencing the accuracy of their evaluation results. With respect to MaAsLin 2 and applications of such simulations to microbiome epidemiology, this was also discussed in the subsection, '**Multivariable association test evaluations**', which further detailed the scope and coverage of our framework, superseding previous benchmarking efforts in several ways:

- As other reviewers pointed out, our benchmark is a clear advance over existing efforts, as it allows for repeated measures and multiple covariates (necessary for nearly all realistic microbiome epidemiology and not considered in tandem by any previous benchmarking efforts).

- Second, it should be pointed out that this manuscript's benchmarking is a secondary outcome of the study, not its primary purpose. The main goal of the research is to provide a validated, integrated framework for accurate microbiome association testing in (mainly) human population studies, which we provide in the form of the MaAsLin 2 model.

- Nevertheless, our benchmarking expands the range of experimental design considerations (more than 1,000 simulated datasets) and the variety of evaluation metrics (more than 10 accuracy measures) compared to previous evaluations. This is detailed in **Response Table 2**, which clearly shows the flexibility and advantage of our framework with respect to previous strategies (**Supplementary Fig. 1B**).

- For the first time, as carefully described in the manuscript, our benchmarking includes more than 84 methods (the largest number of methods and normalization combinations ever tested in this area). To facilitate future methods development, in addition to MaAsLin 2 itself, we also host this benchmarking effort in a separate repository that is aimed at simplifying future method evaluation and reanalysis of published results using new methods along with applications to new datasets: https://github.com/biobakery/maaslin2_benchmark.

- Finally, MaAsLin 2 is the only method in the microbiome literature that can (i) accommodate most common epidemiological study designs, including cross-sectional and longitudinal in a unified framework, (ii) allows joint estimation of multiple groups and time points along with other covariates, and (iii) maintains false discovery rate and power along with providing several data exploration, analysis, and quality control options for the end users (**Response Table 3**), again, completely missed by the reviewer.

  2. Method proposal: As a result of the simulation study the authors propose a particular approach to modeling multi-omics data. The extent of the description is "...a subset or the full complement of metadata are used to model the resulting quality-controlled microbial features and define p-values for each metadata association per feature using one of a wide range of possible multivariate models." This description does not describe a target of inference or a model. What it is doing is made clear in the description of the analysis of the HMP2 data, where random effects models are given in R's notation (L921 p40), indicating that the method is just a wrapper around R's lm and/or lme function. This method is just transforming data, throwing out some rare features, running lm/lme, and doing FDR on lm's p-values. This is not methodological novelty and a workflow paper/blog post could convey the same information more clearly.

Response 3.2 The reviewer may be conflating several independent aspects of our study, which we attempt to clarify below:

- First, if the reviewer's concern is about how evaluated statistical models are applied or tested, we have already provided details of our synthetic benchmarking scheme in the **Methods** section, the main text, and reiterated in summary above. As mentioned before, no previous benchmarking addressed the elements needed for accurate microbiome epidemiology in practice (e.g., false discovery rates, multiple covariates, and repeated measures in tandem) in a unified framework (**Response Table 2**).

- Second, if the reviewer's concern is regarding the MaAsLin 2 implementation, MaAsLin 2 is currently the only method in the literature which implements (i) multiple normalization schemes, (ii) multiple statistical models, (iii) multiple covariates, (iv) multiple fixed and random effects, and (v) multiple in-built visualization and pre-processing options for the users, all while maintaining a good false discovery rate control and reasonable detection power (**Response Table 3**).

- Third, the reviewer seemed to underemphasize the utility of our approach by missing out on several important and essential details. For example, the reviewer simply overlooked the **shuffled data validation** subsection in **Methods**, which is a second robust stream of validations we did before applying any method to the HMP2 dataset (**Supplementary Fig. S13)**. This is in turn also independent from the synthetic evaluation based on SparseDOSSA. In particular, we ruled out the effect of false positive findings using a rigorous permutation-based approach by randomly shuffling the dataset many times, which served as a justification for applying LM and other related models that consistently maintained the false positive rates below the desired 5% significance threshold.

- Finally, the reviewer's underassessment of our validated tool undermines the utility of many practical bioinformatics methods: the novelty of our approach, like many existing tools in the literature, lies in finding an optimized combination of steps to use for a particular problem (here, microbiome epidemiology). MaAsLin 2 is not intended to introduce new statistical theory, but instead to combine several existing steps that, together, are quantitatively better than previous methods and rigorously validated, while providing an informed implementation and convincing applications.

In summary, this paper shows results from a black box simulation study, that is then used to justify proposing a "novel method" that is actually just a wrapper for existing tools.

Response 3.3 We respectfully disagree with Reviewer 3's summary of MaAsLin 2, as the reviewer may have missed several essential components of the method, including:

- First and foremost, the reviewer seems to be over-focused on the HMP2 analysis, whose results were already published independently using MaAsLin 2 (PMID: 31142855). This is apparent from the reviewer's unusual assessment of MaAsLin 2 as a 'wrapper', as it is one of the first implementations in the field to carefully curate appropriate models and normalization schemes for microbiome epidemiology, which is critical in setting standards and establishing best practices in the field, as also acknowledged by Reviewers 1 and 2.

- To further point out the novelty of our framework, MaAsLin 2 is currently the only tool in the literature which implements (i) multiple normalization schemes, (ii) multiple statistical models, (iii) multiple covariates, (iv) multiple fixed and random effects, and (v) multiple in-built visualization and pre-processing options for the users, while also maintaining a good false discovery rate control and sufficient power (**Response Tables 1-3**).

- Finally, the reviewer's criticism on the 'black box' nature of our validation seems implausible, as all three main components of our validation, i.e. (i) the simulation tool (SparseDOSSA as R/Bioconductor package: https://huttenhower.sph.harvard.edu/sparsedossa/), (ii) the benchmarking strategy (https://github.com/biobakery/maaslin2_benchmark), and (iii) the method implementation (MaAsLin 2, again as R/Bioconductor package: https://huttenhower.sph.harvard.edu/maaslin/) are completely reproducible, with open-access source code, documentation and tutorial data available for end users.

Response Table 1: Summary of existing tools for microbiome differential abundance analysis

	DESeq2 edgeR	ZIB	metagenomeSeq	limma limma VOOM	ANCOM ANCOM2	MaAsLin 2
Designed for multiple measurement types (e.g. counts, ratios, and normalized counts)?	No	No	No	No	No	Yes
Implements multiple base models?	No	No	No	No	No	Yes
Implements multiple normalizations?	No	No	No	No	No	Yes
Implements multiple data transformations?	No	No	No	No	No	Yes
Controls FDR?	Yes	No	No	Yes	No	Yes
Controls FPR?	Yes	Yes	No	Yes	Yes	Yes
Handles zero-inflation?	No	Yes	Yes	No	No	Yes
Handles multiple covariates?	Yes	Yes	Yes	Yes	No	Yes
Can fit a fixed effects model?	Yes	No	Yes	Yes	Yes	Yes
Handles repeat measures?	No	Yes	Yes	Yes	Yes	Yes
Handles multiple random effects?	No	No	No	No	No	Yes
In-built QC such as independent filtering?	Yes	No	No	No	No	Yes
In-built visualization?	Yes	No	Yes	Yes	No	Yes

Response Table 2: Summary of existing benchmarking for differential abundance analysis

	Weiss PMID: 28253908	Hawinkel PMID: 28968702	Mcmurdie PMID: 24699258	Thorsen PMID: 27884206	Calgaro PMID: 32746888	Chen PMID: 27187200	MaAsLin 2 SparseDOSSA
Generates realistic synthetic data from template real data?	Yes	Yes	Yes	Yes	Yes	No	Yes
Simulation model	Multinomial	Marginal count model + gaussian copula + resampling	Multinomial	Subsetting /resampling	Subsetting /resampling	Marginal zero-inflated beta	Zero-inflated log-normal + resampling
Able to simulate new microbial observations?	Yes	Yes	Yes	No	No	Yes	Yes
Accounts for zero-inflation?	No	No	No	No	No	Yes	Yes
Accounts for compositionality?	Yes	Yes	Yes	No	No	No	Yes
Accounts for feature-feature association?	No	Yes	No	No	No	No	Yes
Evaluates multiple covariates?	No	No	No	No	No	No	Yes
Incorporates repeated measures?	No	No	No	No	No	Yes	Yes
Incorporates both multiple covariates and repeated measures?	No	No	No	No	No	No	Yes
Incorporates multivariable spike-ins?	No	No	No	No	No	No	Yes
Calculates multiple evaluation metrics beyond FPR, FDR, and power?	No	Yes	No	No	No	No	Yes
Evaluates multiple normalizations?	Yes	Yes	Yes	No	No	No	Yes
Evaluates multiple transformations?	No	No	No	No	No	No	Yes

Response Table 3: Summary of MaAsLin 2 options, modules, and models

MaAsLin 2 functionality as of October, 2020	Options
Input data	Counts<br>Normalized counts<br>Proportions<br>Ratios
Base models (non-zero-inflated)	Linear model<br>Negative Binomial
Base models (zero-inflated)	Compound Poisson<br>Zero-inflated Negative Binomial
Quality control	Variance filtering<br>Prevalence filtering<br>Abundance filtering<br>Covariate standardization
Normalization	TSS<br>RLE<br>CSS<br>TMM<br>NONE
Multiplicity adjustment	Bonferroni<br>BH<br>BY<br>Holm<br>Hommel<br>Hochberg
Transform	AST<br>LOG<br>LOGIT<br>CLR<br>NONE
Output and visualization	Heatmap<br>Scatter plots for continuous metadata<br>Box plots for categorical metadata<br>Association tables<br>Residuals and estimated random effects for further downstream analysis
Software compatibility	R/Bioconductor<br>Command line interface<br>bioBakery Docker
